# Analysis of the Role of Igf2 in Adrenal Tumour Development in Transgenic Mouse Models

**DOI:** 10.1371/journal.pone.0044171

**Published:** 2012-08-28

**Authors:** Coralie Drelon, Annabel Berthon, Bruno Ragazzon, Frédérique Tissier, Roberto Bandiera, Isabelle Sahut-Barnola, Cyrille de Joussineau, Marie Batisse-Lignier, Anne-Marie Lefrançois-Martinez, Jérôme Bertherat, Antoine Martinez, Pierre Val

**Affiliations:** 1 Clermont Université, Université Blaise Pascal, GReD, Clermont-Ferrand, France; 2 CNRS UMR 6293, GReD, Aubière, France; 3 Inserm U1103, GReD, Aubière, France; 4 Institut Cochin, Université Paris Descartes, CNRS UMR 8104, Paris, France; 5 Inserm U1016, Paris, France; 6 Assistance Publique Hôpitaux de Paris, Hôpital Cochin, Department of Endocrinology, Reference Center for Rare Adrenal Diseases, Paris, France; 7 Inserm UMR636, Nice, France; 8 Centre Hospitalier Universitaire, Service d'Endocrinologie, Faculté de Médecine, Clermont-Ferrand, France; Innsbruck Medical University, Austria

## Abstract

Adrenal cortical carcinomas (ACC) are rare but aggressive tumours associated with poor prognosis. The two most frequent alterations in ACC in patients are overexpression of the growth factor IGF2 and constitutive activation of Wnt/β-catenin signalling. Using a transgenic mouse model, we have previously shown that constitutive active β-catenin is a *bona fide* adrenal oncogene. However, although all these mice developed benign adrenal hyperplasia, malignant progression was infrequent, suggesting that secondary genetic events were required for aggressive tumour development. In the present paper, we have tested IGF2 oncogenic properties by developing two distinct transgenic mouse models of Igf2 overexpression in the adrenal cortex. Our analysis shows that despite overexpression levels ranging from 7 (basal) to 87 (ACTH-induced) fold, Igf2 has no tumour initiating potential in the adrenal cortex. However, it induces aberrant accumulation of Gli1 and Pod1-positive progenitor cells, in a hedgehog-independent manner. We have also tested the hypothesis that Igf2 may cooperate with Wnt signalling by mating Igf2 overexpressing lines with mice that express constitutive active β-catenin in the adrenal cortex. We show that the combination of both alterations has no effect on tumour phenotype at stages when β-catenin-induced tumours are benign. However, there is a mild promoting effect at later stages, characterised by increased Weiss score and proliferation. Formation of malignant tumours is nonetheless a rare event, even when Igf2 expression is further increased by ACTH treatment. Altogether these experiments suggest that the growth factor IGF2 is a mild contributor to malignant adrenocortical tumourigenesis.

## Introduction

Adrenocortical carcinomas (ACC) are rare with an incidence of 0.5 to 2 new cases per year and per million [1]. However, these tumours are associated with bad prognosis. Eighty percent of ACC patients develop metastases, which results in five years survival rates of 16 to 47% depending on the series [2]. Most ACC (76%) are also associated with excessive hormone secretion [3], [4]. Clinical management of these cancers mostly relies on resection of primary tumours and mitotane treatment alone [5] or in combination with chemotherapy [6]. However, almost half of the patients present with incurable metastatic disease at diagnosis [2], [7]. Molecular analyses have shown that the two most frequent alterations in ACC are overexpression of Igf2 [8]–[10] and constitutive activation of the Wnt/β-catenin signalling pathway [11]–[13]. We have recently shown that constitutive activation of β-catenin in the adrenal cortex of transgenic ΔCat mice resulted in adrenal hyperplasia and aggressive tumour formation in only a subset of 17 month-old animals [14]. This demonstrated that β-catenin was an adrenal oncogene but it also suggested that secondary genetic alterations were required for malignant progression. IGF2 is a growth factor involved in the control of cell proliferation and inhibition of apoptosis. The IGF2 gene locus at 11p15 is under the control of parental epigenetic imprinting. Loss of imprinting (LOI) at 11p15 causes Beckwith-Wiedmann syndrome (BWS) characterised by embryonic overgrowth and predisposition to tumour development, among which adrenocortical carcinomas [15]. In sporadic adrenal tumours, IGF2 is overexpressed in 80–90% of ACC but not in adrenocortical adenomas (ACA) [9], [16]. In most cases, IGF2 overexpression results from loss of the maternal IGF2 allele and duplication of the paternal allele (paternal unidisomy) but it is also associated with alterations in epigenetic imprinting at 11p15 [8]. Other components of the IGF signalling pathway such as the IGFIR receptor [17] and IGF binding protein 2 (IGFBP2) [18] are also overexpressed in ACC. Altogether these observations suggest that overexpression of IGF2 could be involved in adrenal tumourigenesis. Consistent with this idea, IGF1R antagonists can inhibit proliferation of the human adrenocortical carcinoma H295R cell line both in culture and in xenograft experiments [19], [20].

Mouse models of increased Igf2 signalling recapitulate most of the developmental and growth abnormalities found in BWS, but fail to develop adrenal hyperplasia or tumours [21], [22]. Transgenic mice with overexpression of human IGF2 under the control of the PEPCK promoter show 4 to 6 fold elevation of serum IGF2 levels and moderate transgene expression in the adrenal cortex. This results in mild adrenocortical hyperplasia characterised by a 1.5 fold increase in zona fasciculata volume [23]. However, these mice did not develop adrenocortical tumours over the 18 months time course of the study. This suggested that Igf2 overexpression might not initiate adrenal tumour development but that it could be involved in malignant progression.

In order to test this hypothesis, we have developed two transgenic mouse models in which mouse Igf2 is specifically overexpressed in the adrenal cortex through the regulatory regions of the Akr1b7 or the P450SCC gene, which allow basal and ACTH stimulated transgene expression. We have also engineered a mouse model in which the two most frequent genetic alterations found in ACC patients were combined by mating Igf2 overexpressing mice with mice that express a constitutive active β-catenin in the adrenal cortex (ΔCat mice) [14].

Our analysis of these models shows that Igf2 overexpression does not induce adrenal tumour development although it triggers aberrant recruitment of adrenal progenitors cells in a hedgehog independent manner. We also show that Igf2 overexpression in the context of constitutive β-catenin activation only has a mild promoting effect on tumour progression. Altogether, these data strongly suggest that despite marked overexpression in adrenal carcinomas, Igf2 is unlikely to play a major role in either tumour initiation or progression in the adrenal cortex.

## Materials and Methods

### Ethics statement

All animal studies were approved by Auvergne Ethics Committee and were conducted in agreement with international standards for animal welfare in order to minimize animal suffering.

### Construction of the transgenes

The 0.5 Akr1b7:Igf2 transgene was constructed by inserting the cDNA of mouse Igf2 into the 0.5 Akr1b7-CAT-int backbone. The 0.5Akr1b7-CAT-Int construct contains the −510/+44 fragment of the mouse Akr1b7 gene fused to a reporter gene composed of the CAT coding sequence followed by the mini-intron and polyadenylation signal from SV40 T antigen sequences. These sequences are followed by 3.5 kb of an intragenic fragment spanning intron 1 to 2 of the Akr1b7 gene. The CAT reporter gene was excised from the 0.5 Akr1b7-CAT-Int transgene by a NcoI digestion. This was followed by a fill-in of the cohesive ends by Klenow polymerase and subsequent XhoI digestion. The complete mouse Igf2 cDNA (positions 1126 to 1668 on sequence NM_010514.2) was amplified from mouse liver mRNAs using primers Igf2-Fwd-5′-CCCTCGAGACCATGGGGATCCCAGTG-3′ and Igf2-Rev-5′-AAAGTACTTTTTCACTGATGGTTGCTGGAC-3′. These primers contained XhoI (Fwd) and ScaI (Rev) restriction sites that were included in the PCR fragment. After digestion with XhoI and ScaI, the PCR fragment was cloned into the XhoI and blunted NcoI sites of the 0.5 Akr1b7-Int construct.

The 4.5 Scc:Igf2 transgene was constructed by cloning the regulatory sequences of the human P450SCC gene and mouse Igf2 cDNA in the pBL-CAT3 backbone. For this, the CAT gene was excised from the pBLCAT3 reporter plasmid by a NcoI digestion. This was followed by a fill-in of the cohesive ends by Klenow polymerase and subsequent XhoI digestion. The mouse Igf2 cDNA was the inserted in this plasmid as described above, resulting in the pBL-Igf2 construction. Regulatory regions from the human P450SSC gene (−4400/+55) were amplified from human genomic DNA using primers SCC-Fwd-5′-GCTCTAGAGCGACCCCTCCCAAGGCCAAACAAA-3′ and SCC-Rev-5′-CCCTCGAGGGCCCACAGCTGTGACTGTAC-3′. These contained an XbaI (Fwd) and an XhoI (Rev) sequence. The PCR product was digested by XhoI and XbaI. It was subsequently cloned into pBL-Igf2 after an XhoI/XbaI digestion.

### Mice

All animal studies were approved by Auvergne Ethics Committee and were conducted in agreement with international standards for animal welfare. Mice were culled by decapitation at the end of experiments. Tissues were collected, frozen in liquid nitrogen and stored at −80°C.

### Generation of transgenic mice

Transgenic mice were generated by additive transgenesis. Before micro-injection, the 0.5 Akr1b7:Igf2 and 4.5 Scc:Igf2 transgenes were excised by KpnI/HindIII and EcoRI/SalI digestions, respectively. The resulting linearized fragment was injected into fertilised B6D2 mouse oocytes. Detection of transgenic founders and determination of copy numbers were performed by southern blotting. Eight and three founders were generated for the 0.5 Akr1b7:Igf2 and 4.5 Scc:Igf2 lines, respectively (Figure S1). Founders F0–10, F0–20 and F0–22 transmitted the 0.5 Akr1b7:Igf2 transgene to their offspring. Founders F0–14 and F0–19 transmitted the 4.5 Scc:Igf2 transgene. Semi quantitative RT-PCR analyses were used to select the lines with the most specific expression pattern and highest expression levels in the adrenal gland. Lines F0–20 (0.5 Akr1b7:Igf2) and F0–19 (4.5 Scc:Igf2) were selected for further experiments. The line bearing the 0.5 Akr1b7:Igf2 transgene will be referred to as AdIgf2 throughout this manuscript. The line bearing the 4.5 Scc:Igf2 transgene will be referred to as Scc:Igf2. ΔCat mice were previously described [24]. They were generated by mating Catnblox^(ex3)^ mice [25] with 0.5 akr1b7:Cre mice (24). ΔCat;AdIgf2 and ΔCat;Scc:Igf2 lines were generated by mating ΔCat mice with AdIGF2 and Scc:Igf2 transgenic lines respectively.

### Treatment with ACTH

Twelve month-old mice were treated chronically with intramuscular injections of 1.2 units of Synacthene Retard (tetracosactide complexed with zinc chloride, Sigma-Tau Laboratories) every other day, for two months.

### Reverse-transcription quantitative PCR

Frozen tissues were disrupted in Nucleospin RNA II lysis buffer (Macherey Nagel) using the Tissue-Lyser system (Qiagen). Total mRNAs were extracted using the NucleoSpin RNA II kit, according to manufacturer's instructions. Five hundred ng of mRNAs were reverse transcribed for 1 hour at 42°C with 5 pmol of random hexamer primers (U1240; Promega), 200 units reverse transcriptase (M1701; Promega), 2mM dNTPs and 20 units RNAsin (N2615; Promega). Igf1r and 36b4 were amplified by standard PCR with 1.5 units of GoTaq polymerase (M8305; Promega), 2mM of MgCl2, 2.5mM of dNTPs and 10pmol of forward and reverse primers (Igf1R fwd-5′ GAGAAATTTGTGGGCCCGGCATTGA and rev -5′GCATCCAAGATGAGAGACCAGTCTA; 36b4 fwd-5′ GTCACTGTGCCAGCTCAGAA and rev-5′ TCAATGGTGCCTCTGGAGAT). Igf1R amplification was carried out using the following conditions: 94°C, 30 s; 68°C, 30s; 72°C, 30s for a total of 30 cycles. 36b4 was amplified using the following protocol: 94°C, 30 s; 60°C, 30s; 72°C, 30 s for 30 cycles. Gene expression levels were measured by real-time quantitative PCR (RTqPCR) using either probes from the Taqman gene expression assays pool (Aplied Biosystems) or SYBR green and standard PCR primers. In either case, 2 µl of diluted cDNAs (1/40) were used as a template in each PCR reaction. For Taqman analyses, each reaction was performed in duplicate in a final volume of 15µl with 0.75 µl of the appropriate probe mix and 7.5µl of PCR Mastermix (Precision, Ic., Primerdesign.co.uk). For SYBR Green analyses, each reaction was performed in duplicate in a final volume of 25µl with 12.5µl of MESA Green qPCR mix (Eurogentec) and 10 pmol of forward and reverse primer. Relative mRNA accumulation was determined by the ΔΔCt method with *peptidylprolyl isomerase B* (*ppib*) as a standard for Taqman analyses and with *36b4* as a standard for SYBR Green analyses. Unless otherwise stated, statistical analysis was performed with Student's *t*-test. Taqman gene expression assay probes that were used in the study are: Ppib Mm00478295_m1, Gli1 Mm004494645_m1, Patched1 Mm00436026_m1, Pod1 Mm00448961_m1, Shh Mm00436527_m1, Axin2 Mm00443610_m1, Lef-1 mM00550265_m1, Vegf-A Mm01281449_m1, Nov/Ccn3 Mm00456855_m1, Connexin-α43 (Gja1) Mm00439105_m1. Primer sequences for genes studied with SYBR Green are: 36b4 Fwd-5′ GTCACTGTGCCAGCTCAGAA and 36b4 Rev: 5' TCAATGGTGCCTCTGGAGAT; Cyclin D1 Fwd: 5' TCTCCTGCTACCGCACAAC and Cylin D1 Rev: 5' TTCCTCCACTTCCCCCTC.

### Histology and immuno-histochemistry

Adrenals were fixed in 4% paraformaldehyde overnight. After two washes in PBS, adrenals were dehydrated through an ethanol gradient and incubated for two hours in Histoclear (HS200; National diagnostics). They were then embedded in paraffin and 5 µm sections were cut. For evaluation of general morphology, sections were stained with haematoxylin and eosin on a Microm HMS70 automated processor (Microm Microtech, France), according to standard procedures. Immunodection of Igf2 1/100 (ab9574, Abcam), phospho-S6 1/250 (2217, Cell signaling), phospho-S6Kinase 1/100 (Ab32359, Abcam), GATA4 1/50 (SC1237, Santa Cruz), β-catenin 1/1000 (610153, BD Biosciences Pharmingen) Dab2 1/1000 (610464, BD Biosciences), Akr1b7 1/200 [26], Sf-1 1/1000 (a kind gift of Dr Ken Morohashi, Fukuoka, Japan) and Ki-67 1/1000 (RM-9106, Thermo Fisher Scientific) were carried out on an Intavis InSitu Pro VSi automated processor (Intavis AG, Germany).

For immunohistochemical analyses, antigen retrieval was performed by boiling rehydrated sections in sodium citrate 10 mM pH 6 tween 0.05% for 20 min, with the exception of Sf-1 and GATA4, which were detected after 20 min boiling in Vector Antigen Retrieval Solution (H3300, Vector Labs). Primary antibodies were detected with the appropriate secondary antibodies, coupled to biotin (1/500, Jackson Immunoresearch). Biotin was then complexed with streptavidin coupled to Horse Radish Peroxidase (HRP) (016–030–084, Jackson Immunoresearch). HRP activity was detected with the chromogenic substrate Novared (SK4800, Vector Labs). The double staining for Dab2 and Akr1b7 was obtained following a similar protocol. The two proteins were sequentially detected by amplification with TSA-Alexa555 (Molecular Probes) and TSA-Alexa488 (Molecular Probes) flurorescent HRP substrates. To avoid cross-reaction, HRP was inactivated by incubation with 0.02% HCl for 20 min after detection of the first antibody.

### In situ hybridisation

Paraffin-embedded tissues were cut as 10 µm sections on Superfrost Plus slides. In situ hybridisation was conducted according to a standard protocol [27].

### Evaluation of cell proliferation

In smaller adrenals, Ki67-positive cells were counted on the entire adrenal surface. The number of cells was corrected to section surface and was expressed as a percentage of one of the wild-type control adrenals. Counting was performed separately in the cortex and medulla. Results represent the mean of at least 6 individual counts ± standard deviation. In larger tumours, proliferation index was evaluated by counting Ki67-positive cells in five high power field pictures (x40 magnification) for each individual tumour. This was then expressed as a percentage of the total cell count for each field. For each individual adrenal, results were expressed as the mean of counts in five independent fields.

## Results

In order to induce overexpression of Igf2 in steroidogenic cells of the adrenal cortex we developed transgenic mice in which mouse Igf2 expression was driven by 500 bp of the regulatory regions and 3.5 kb of the intronic regions of the Akr1b7 gene (Figure S1A). This regulatory element has been previously shown to induce transgene expression in steroidogenic cells of the cortex starting at E14.5 [28]. Three transgenic lines differing by the genomic insertion site and copy numbers were generated (Figure S1B). Line 20, which showed highest expression in the adrenal by RT PCR (data not shown) was kept for further studies. It will thereafter be referred to as AdIgf2. We also generated a second transgenic line in which Igf2 expression was driven by 4.5 kb of the regulatory regions of the human P450SCC gene. This transgene allows expression from E11.5 in steroidogenic cells of both the adrenal cortex and the gonad [29]. Out of the two established lines, we selected line 19 in which expression in the adrenal was highest (Figure S1B and data not shown). These mice will be referred to as Scc:Igf2 line. They have been used as a control in order to confirm that the phenotypes observed in AdIgf2 adrenals were actually resulting from Igf2 overexpression and not from transgene insertion effects. The core of the experiments in the present manuscript has been conducted with females from the AdIgf2 transgenic line.

**Figure 1:**
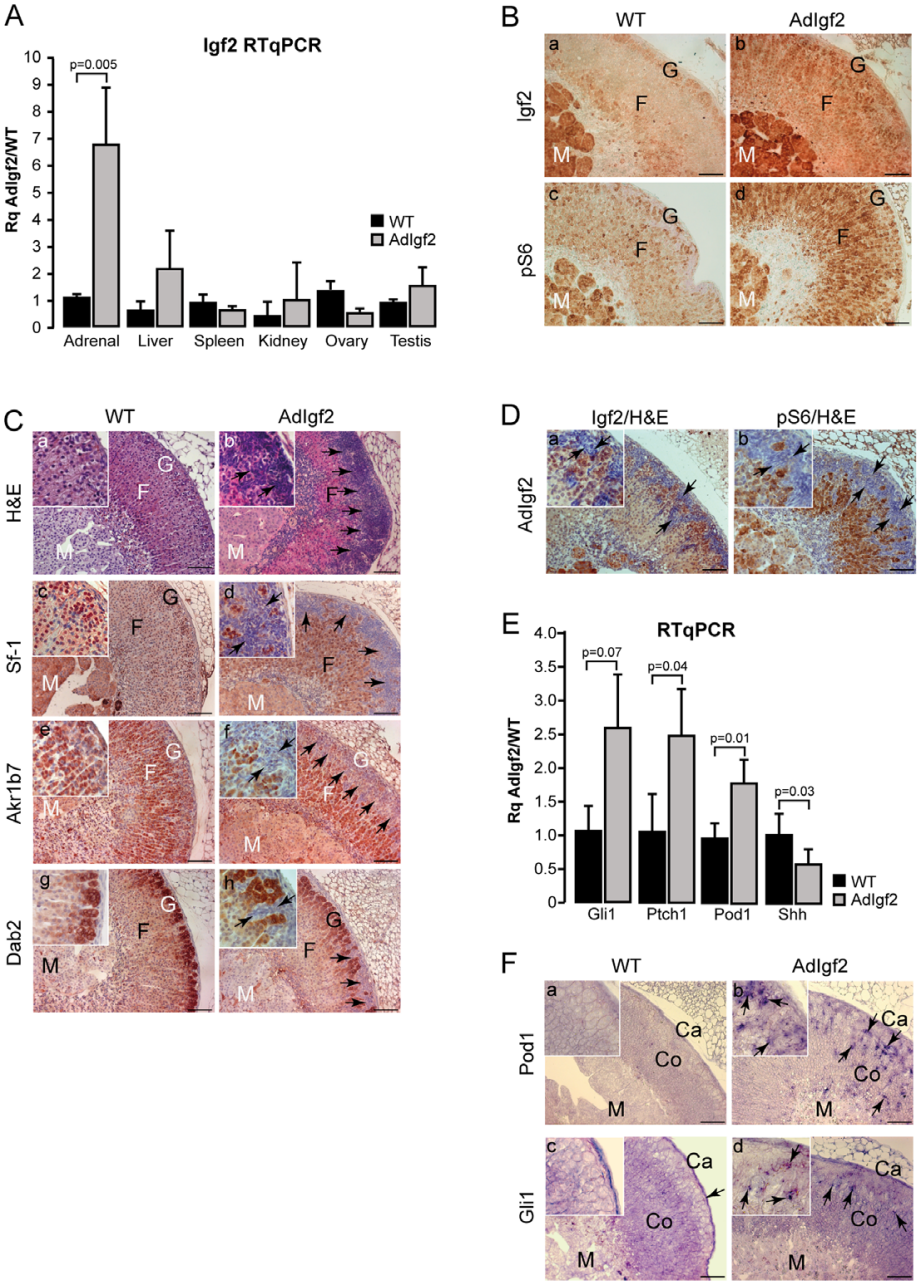
Characterisation of AdIgf2 transgenic mice adrenals. A- Igf2 is overexpressed in the adrenals of transgenic mice. Igf2 expression was analysed by RTqPCR on cDNAs from adrenals, kidneys, liver, spleen, ovaries and testes of 10 month-old wild-type (WT) and AdIgf2 transgenic mice. Bars represent the mean relative quantification (Rq AdIgf2/WT) of Igf2 expression for each tissue in at least 4 samples per genotype ± standard deviation. *P*-value was calculated using Student's *t*-test. **B- Igf2 signalling is increased in AdIgf2 adrenals.** Expression of Igf2 (a–b) and of the phosphorylated ribosomal protein S6 (c–d) was analysed by immunohistochemistry in WT and AdIgf2 adrenals. **C- Effect of Igf2 overexpression on adrenal histology and differentiation.** Histology was analysed by haematoxylin & eosin staining in wild-type (a) and AdIgf2 (b) adrenals. Expression of Sf-1 (c–d), Akr1b7 (e–f) and Dab2 (g–h) was analysed by immunohistochemistry in WT and AdIgf2 adrenals. Black arrows show infiltrating mesenchymal subcapsular cells. **D- Igf2 and phospho-S6 are not expressed in subcapuslar mesenchymal cells.** Igf2 (a) and phospho-S6 (b) were immunodetected in AdIgf2 adrenals and the tissue was counterstained with haematoxylin. **E- Adrenal progenitor cells markers are overexpressed in AdIgf2 adrenal.** Expression of the progenitor cells markers, Gli1, Ptch1, Pod1 and Shh was analysed by RTqPCR on cDNAs from wild-type (WT) and AdIgf2 adrenals. Bars represent the mean relative quantification (Rq AdIgf2/WT) of gene expression for each marker in at least 6 adrenals per genotype ± standard deviation. *P*-value was calculated using Student's *t*-test. **F- Pod1 and Gli1 are overexpressed in AdIgf2 adrenals.** Pod1 and Gli1 expression domains were analysed by in-situ hybridisation in wild-type (a, c) and AdIgf2 (b, d) adrenals. In all panels, arrows show mesenchymal subcapsular cells. M, medulla; F, fasciculata; G, glomerulosa; Co, cortex; Ca, capsule. Scale bar is 80 µm.

Further characterisation of the AdIgf2 transgenic line by RTqPCR showed a 6.8-fold increase in Igf2 in the adrenal cortex of transgenics compared with WT (Figure 1A). There was no significant overexpression in the other tissues tested, confirming the specificity of transgene activity. Igf2 expression was then assessed in situ by immunohistochemistry. Igf2 was barely expressed in the WT adrenal cortex (Figure 1B, a). In contrast, the protein was detected throughout the cortex, in virtually all steroidogenic cells of AdIgf2 adrenals (Figure 1B, b). Igf2 signals by binding to the Igf1-receptor and activates both the PI3K and MAPK pathways. RT-PCR showed expression of similar levels of Igf1r in WT and AdIgf2 adrenals (Figure S2A). We then assessed the ability of Igf2 to induce signalling events in the adrenal cortex by analysing expression of the phosphorylated S6 protein. These experiments showed a basal level of S6 phosphorylation in WT adrenals with few cortical cells exhibiting cytoplasmic staining (Figure 1B, c). In contrast, AdIgf2 adrenals showed strong S6 phosphorylation in almost all steroidogenic cells of the cortex (Figure 1B, d). Similar observations were made for the upstream phosphorylated-S6Kinase (Figure S2B). Altogether, these experiments showed that AdIgf2 transgenic mice overexpressed Igf2, which resulted in increased Igf2 signalling in the adrenal cortex.

The effect of Igf2 overexpression on cortical organisation was then assessed by haematoxylin/eosin staining on sections from 10 month-old AdIgf2 adrenals. These experiments showed that the overall cord-like structure found in the zona fasciculata, was preserved in AdIgf2 adrenals (Figure 1C, b) compared with wild-type tissue (Figure 1C, a). However AdIgf2 adrenals were characterised by accumulation of large amounts of small basophilic cells at the periphery of the cortex, which in the most extreme cases, almost covered a third of cortical surface (Figure 1C, b black arrows). Most of these cells were elongated with scarce cytoplasm and resembled mesenchymal capsular cells (Figure 1C, b inset). Analysis of steroidogenic factor 1 (Sf–1, Figure 1C, d) and Akr1b7 (Figure 1C, f) expression showed that fasciculata cells maintained their molecular identity. Interestingly, the basophilic cells did not express Sf-1 (Figure 1C, d inset) or Akr1b7 (Figure 1C, f inset), indicating that they were undifferentiated. However, some but not all of these cells, expressed the transcription factor GATA4 (Figure S2 C, b) which was almost undetectable in wild-type adrenals (Figure S2 C, a). This was reminiscent of the spindle-shaped cells that accumulate at the cortical periphery in some strains of mice at the initiation of castration-induced neoplasia [30]. Disabled-2 (Dab2), a recently identified molecular marker of zona glomerulosa differentiation [31] was expressed under the capsule of wild-type adrenals (Figure 1C, g). In AdIgf2 adrenals, Dab2 was still expressed in the outer cortex. However, Dab2 expression domain was displaced inwards by accumulation of the undifferentiated basophilic cells (Figure 1C, h) that were negative for Dab2 expression (Figure 1B, h inset). Co-immunohistochemistry for Dab2 and Akr1b7 showed that the relative positions of zona glomerulosa (Dab2) and zona fasciculata (Akr1b7) were unaltered by Igf2 overexpression (Figure S2D). Altogether, these experiments suggested that Igf2 overexpression did not alter cortical architecture but that it induced accumulation of undifferentiated basophilic subcapsular cells. Absence of Igf2 (Figure 1D, a, black arrows) expression and S6 phosphorylation (Figure 1D, b, black arrows) in these cells suggested that their accumulation was triggered by indirect mechanisms that did not involve the PI3K/S6K pathway. Alternatively, this may reflect involvement of this pathway in the recruitment of these cells at an earlier time-point.

King and colleagues recently showed that adrenal cortex renewal occurred through recruitment of subcapsular progenitor cells in response to Shh signalling. Progenitors were undifferentiated (Sf-1 negative) and characterised by expression of Patched1 and Gli1. Shh was expressed by poorly differentiated Sf-1-positive cells localised in the vicinity of the zona glomerulosa [32]. The localisation, histological and molecular characteristics of the supernumerary mesenchymal subcapsular cells in AdIGF2 adrenals suggested that they could represent abnormally accumulated progenitors. Indeed, RTqPCR analysis showed markedly increased expression of both Gli1 and Ptch1 in 10 month-old AdIgf2 compared with wild-type adrenals (Figure 1E). Pod1, another putative marker of adrenal progenitor cells was also significantly upregulated in AdIgf2 adrenals (Figure 1E). These observations were further substantiated by the presence of increased Pod1 and to a lesser extent Gli1 in situ hybridisation staining in the outer cortex and capsule of AdIgf2 adrenals (Figure 1F, b, d) compared with wild-type (Figure 1F, a, c). Surprisingly though, Shh expression was significantly decreased (Figure 1E), suggesting that accumulation of progenitors in response to Igf2 overexpression was independent of Hedgehog signalling.

In order to confirm that these effects were strictly dependent on Igf2 over-expression, similar experiments were performed on Scc:Igf2 transgenic mice in which Igf2 expression was driven by P450SCC regulatory regions (Figure S1). RTqPCR analysis showed that Scc:Igf2 mice expressed levels of Igf2 that were slightly higher than in AdIgf2 adrenals (10–11 fold Figure S3A). Igf2 overexpression through P450SCC regulatory regions also induced phosphorylation of S6 (Figure S3B) and resulted in accumulation of progenitor cells albeit at a later timepoint (Figure S3C&D). Altogether these data showed that Igf2 overexpression in the adrenal cortex of transgenic mice did not alter adrenal architecture but induced accumulation of undifferentiated progenitor cells, independently of Shh expression.

**Figure 2:**
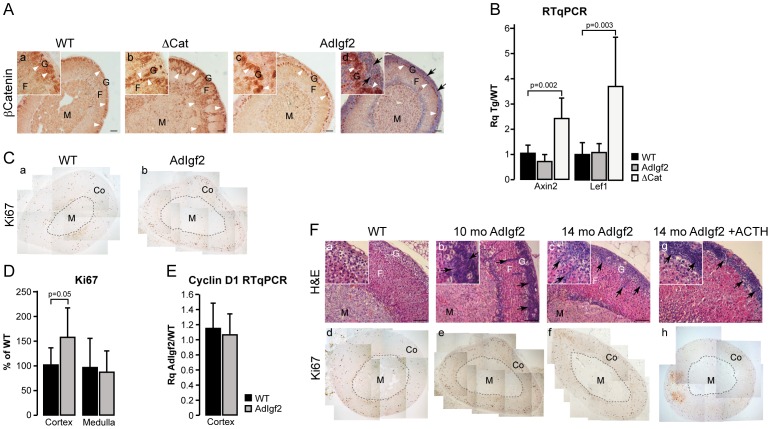
Igf2 overexpression does not initiate adrenal tumourigenesis. A- β-catenin is not activated in AdIgf2 adrenals. β-catenin expression was analysed by immunohistochemistry in wild-type (WT, a), ΔCat (positive control, b) and AdIgf2 (c–d) adrenals. Section in c was counterstained with haemotxylin. Nucleo-cytoplasmic staining of β-catenin (white arrowheads) is restricted to zona glomerulosa in WT and AdIgf2 adrenals but spreads throughout the cortex and inside the medulla in ΔCat adrenals. Black arrows show infiltrating mesenchymal subcapsular cells. **B -Wnt pathway is not activated in AdIgf2 adrenals.** Expression levels of Axin2 and Lef-1, two canonical Wnt pathway targets, were determined by RTqPCR with cDNAs from wild-type, AdIgf2 and ΔCat (positive control) adrenals. Bars represent the mean relative quantification (Rq Tg/WT) of gene expression for each gene in at least 6 adrenals per genotype ± standard deviation. *P*-value was calculated using Student's *t*-test. **C- Analysis of Ki67 expression.** Ki67 was detected by immunohistochemistry on wild-type (WT, a) and AdIgf2 adrenals (b). **D-** **Proliferation is not increased in AdIgf2 adrenals.** Numbers of Ki67-positive cells in a whole adrenal section were counted separately in the cortex (Co) and medulla (M). For each zone, the number of cells was corrected for surface and is expressed as a percentage of Ki67-positive cells in one of the control individuals. Bars represent the mean of at least 7 individual counts in wild-type and AdIgf2 adrenals ± standard deviation. *P*-value was calculated using Student's *t*-test. **E- Cyclin D1 expression is not increased in AdIgf2 adrenals.** Expression of Cyclin D1 was analysed by RTqPCR with cDNAs from Wild-type and AdIgf2 adrenals. Bars represent the mean relative quantification (Rq AdIgf2/WT) of gene expression in at least 5 adrenals per genotype ± standard deviation. *P*-value was calculated using Student's *t*-test. **F- Igf2 overexpression does not induce adrenal tumour formation.** The phenotype of AdIgf2 adrenals was followed over a 14 months time course by haematoxylin & eosin staining (b–c) and immunohistochemistry for Ki67 (e–f). A 10 month-old wild-type adrenal was included as a reference (WT, a, d). Some twelve month-old AdIgf2 mice were treated with ACTH for two months in order to increase Igf2 expression. Adrenal histology and proliferation were then assessed by haematoxylin & eosin staining (g) and Ki67 immunohistochemistry (h). Arrows show mesenchymal subcapsular cells. M, medulla; F, fasciculata; G, glomerulosa; Co, cortex. Scale bar is 80 µm.

Molecular analyses on cell culture models suggested that Igf2 signalling could be involved in the stimulation of canonical Wnt/β-catenin signalling. This crosstalk was assumed to play a role in the potential oncogenic effects of Igf2 [33]–[37]. We have recently shown that β-catenin is an adrenal oncogene involved in tumour initiation in the adrenal cortex [14]. Therefore, we wanted to investigate a potential involvement of Igf2 in Wnt signalling stimulation in the adrenal cortex. Nucleo-cytoplasmic staining of β-catenin is the gold standard in histological evaluation of canonical Wnt signalling. We thus analysed β-catenin expression by immunohistochemistry (Figure 2A). Adrenals from ΔCat mice in which β-catenin is constitutively activated were included as a control [14]. In the wild-type adrenal, nucleo-cytoplasmic β-catenin accumulation was restricted to zona glomerulosa (Figure 2A, a inset, white arrowheads). As previously published [14], ΔCat adrenals showed nucleo-cytoplasmic β-catenin staining throughout the cortex and within steroidogenic cells that invaded the medulla (Figure 2A, b inset). In contrast, nucleo-cytoplasmic β-catenin staining was restricted to zona glomerulosa in AdIgf2 adrenals (Figure 2A, c inset), although Igf2 was overexpressed throughout the cortex (Figure 1B, b). As shown in Figure 1, zona glomerulosa was displaced inwards by the accumulation of basophilic subcapsular cells (Figure 2A, d inset). Altogether, these observations suggested that Igf2 overexpression did not cause canonical Wnt pathway stimulation in the adrenal cortex. In order to confirm this hypothesis, we analysed expression of two β-catenin target genes by RTqPCR. As expected, expression levels of both Axin2 and Lef1 were markedly increased in ΔCat adrenals (Figure 2B). In contrast, overexpression of Igf2 did not alter expression of either target genes (Figure 2B). We thus concluded that Igf2 overexpression did not result in canonical Wnt signalling stimulation in the adrenal cortex.

IGF2 overexpression is found in almost all adrenal carcinomas compared with adrenal adenomas or healthy adrenals [8], [9]. This suggests that Igf2 may be involved in carcinogenic transformation in the adrenal. We thus evaluated proliferation in 10 month-old AdIgf2 adrenals by counting the number of cells expressing Ki67 (Figure 2C). These experiments showed a modest but significant increase in Ki67-positive cells in the cortex of AdIgf2 mice compared with wild-type adrenals, whereas proliferation was unaltered in the medulla (Figure 2D). Increased Ki67 staining was randomly distributed throughout the cortex and was not associated with accumulating progenitor cells (data not shown). This modest increase in proliferation was not associated with increased Cyclin D1 expression as measured by RTqPCR, which was in agreement with the absence of Wnt/β-catenin signalling stimulation (Figure 2E). Analysis of adrenal histology (Figure 2F, a–c) and Ki67 expression (Figure 2F, d–f) in 14 month-old adrenals showed that there was no progression of the adrenal phenotype over time. These observations suggested that Igf2 overexpression was not sufficient to trigger adrenal cortex tumourigenesis. However, Igf2 overexpression in this setup was mild (6.8 fold) compared to overexpression in ACC patients (from 10 to 1000 fold). We thus took advantage of the sensitivity to ACTH of the transgenic construct to increase Igf2 expression. For these experiments, 12 month-old AdIgf2 mice were treated three times a week with 1.2 units of long acting ACTH for two months. This resulted in an 87 ± 4 fold increase in Igf2 expression as measured by RTqPCR (Figure S3E). As expected, chronic ACTH exposure caused marked hypertrophy of cortical cells (Figure 2F, g) compared with both wild-type (Figure 2F, a) and untreated AdIgf2 adrenals (Figure 2F, b–c). However, the marked increase in Igf2 expression had no obvious effect on either cortical organisation (Figure 2F, g) or proliferation as measured by Ki67 expression (Figure 2F, h). Altogether, these data strongly suggested that Igf2 overexpression alone could not trigger tumour development in the adrenal cortex.

**Figure 3:**
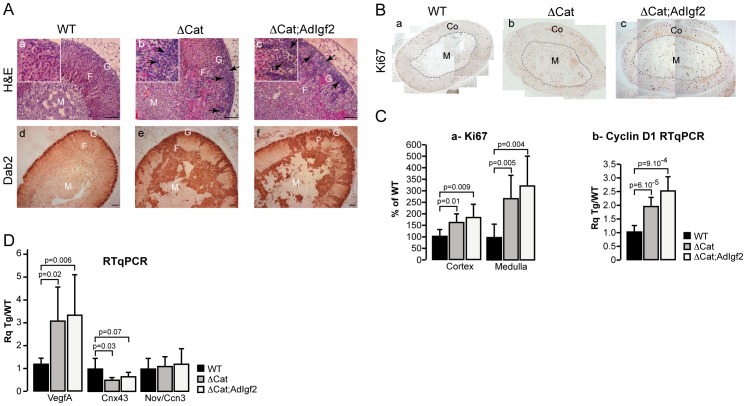
Analysis of the cooperation between β-catenin activation and Igf2 overexpression in 10 month-old mice. A- Igf2 overexpression in 10 month-old ΔCat mice does not significantly alter the adrenal phenotype. AdIgf2 mice were mated with ΔCat mice to generate ΔCat;AdIgf2 compound animals. The adrenal phenotype in 10 month-old mice was analysed by haematoxylin & eosin staining (a–c) and immunohistochemistry for Dab2. Black arrows show infiltrating subcapsular mesenchymal cells. **B- Analysis of Ki67 expression in 10 month-old ΔCat;AdIgf2 adrenals.** Ki67 expression was analysed by immunohistochemistry in wild-type (B, a), ΔCat (B, b) and ΔCat;Igf2 (B, c) adrenals. **C- Igf2 overexpression in ΔCat mice does not significantly increase proliferation. a-Counting of Ki67-positive cells.** Numbers of Ki67-positive cells in a whole adrenal section were counted separately in the cortex (Co) and medulla (M). For each zone, the number of cells was corrected for surface and is expressed as a percentage of Ki67-positive cells in one of the control individuals. Bars represent the mean of at least 7 individual counts in wild-type, ΔCat and ΔCat;AdIgf2 adrenals ± standard deviation. *P*-value was calculated using Student's *t*-test. **b- Analysis of Cyclin D1 expression.** Cyclin D1 expression was analysed by RTqPCR with cDNAs from wild-type, ΔCat and ΔCat;AdIgf2 adrenals. Bars represent the mean relative quantification (Rq Tg/WT) of gene expression in at least 7 adrenals per genotype ± standard deviation. *P*-value was calculated using Student's *t*-test. **D- Analysis of malignancy markers expression.** Expression of VegfA, Connexina43 (cnx43) and Nov/Ccn3 was analysed by RTqPCR with cDNAs from wild-type (WT), ΔCat and ΔCat;AdIgf2 adrenals. Bars represent the mean relative quantification (Rq Tg/WT) of gene expression for each marker in at least 7 adrenals per genotype ± standard deviation. *P*-value was calculated using Student's *t*-test. M, medulla; F, fasciculata; G, glomerulosa. Scale bar is 80 µm.

In ACC patients, IGF2 overexpression is frequently associated with constitutive activation of β-catenin. This suggests that alterations in both pathways may accelerate malignant tumour progression. We thus tested this hypothesis by mating AdIgf2 mice with ΔCat mice to generate ΔCat;AdIgf2 compound transgenics. Our initial analysis focused on 10 month-old mice for which we previously generated most of our data. As expected, constitutive activation of β-catenin through deletion of the third exon of the Ctnnb1 gene, resulted in benign adrenal hyperplasia, accumulation of basophilic sub-capsular cells (Figure 3A, b inset, black arrows) and ectopic differentiation of zona glomerulosa cells, shown by expansion of the domain of Disabled2 (Dab2) expression throughout the cortex and inside the medulla (Figure 3A, e). Surprisingly, overexpression of Igf2 in the context of constitutive β-catenin activation did not significantly alter adrenal histology (Figure 3A, c) or Dab2 expression (Figure 3A, f and Figure S4) in the majority of double transgenics when compared with ΔCat mice. The moderate effect of Igf2 overexpression in a ΔCat context was also observed by histological analysis of compound transgenics in which Igf2 was overexpressed through P450SCC regulatory regions (ΔCat;Scc:Igf2, Figure S5). Analysis of the proliferative parameters by Ki67 staining, showed that the number of proliferating cells was significantly increased in the cortex and medulla of ΔCat (Figure 3B, b & Figure 3C, a) and ΔCat;AdIgf2 (Figure 3B, c & Figure 3C, a) adrenals when compared with wild-type (Figure 3B, a & Figure 3C, a). However there was no significant difference between the two genotypes (Figure 3B & C, a). This was further corroborated by RTqPCR analysis of Cyclin D1 expression (Figure 3C, b). Altogether, these experiments suggested that there was no major acceleration of tumour development upon overexpression of Igf2 in the context of constitutive β-catenin activation in 10 month-old adrenals. In order to confirm these observations, we analysed expression of three markers associated with malignancy in ACC patients (Figure 3D). Expression of Vegf-A was significantly increased in both ΔCat and ΔCat; AdIgf2 adrenals. However, there was no further significant increase in ΔCat; AdIgf2 when compared with ΔCat adrenals. Absence of an additive effect of Igf2 was also confirmed for connexin-alpha 43 (Cnx43) for which there was a similar down-regulation in both genotypes. In contrast with our previous analysis of 18 month-old ΔCat adrenals [14], we did not observe an alteration of Nov/Ccn3 expression upon constitutive activation of β-catenin in 10 month-old mice. Again, overexpression of Igf2 had no impact on Nov/Ccn3 expression (Figure 3D). Collectively, these data suggested that Igf2 did not cooperate with β-catenin in the early steps of adrenal cortex tumourigenesis.

**Figure 4:**
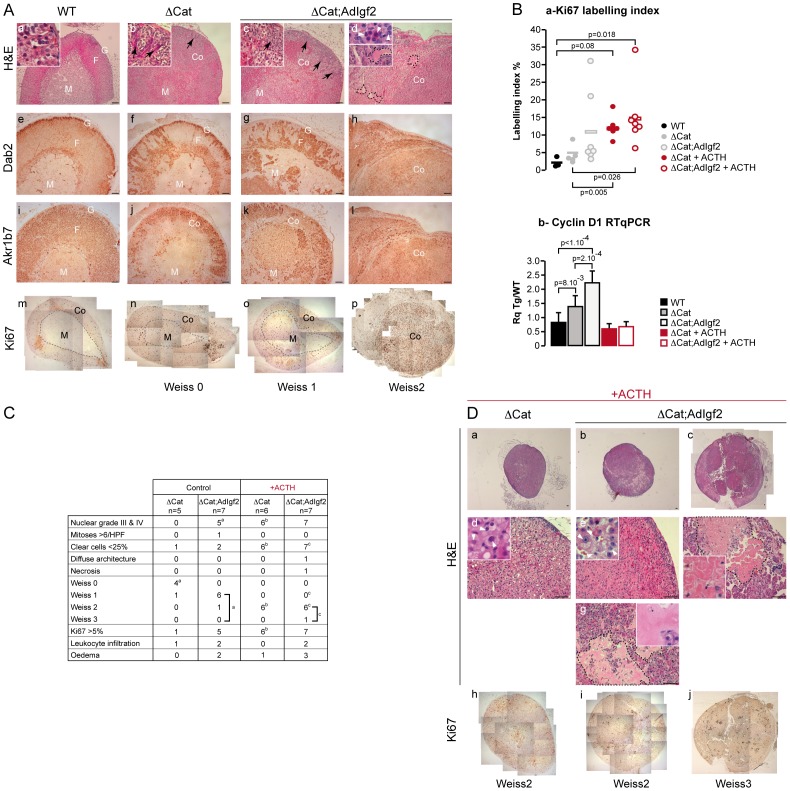
Overexpression of Igf2 results in a mild acceleration of tumour progression in 14 month-old ΔCat adrenals. A- Histological analysis of the adrenal phenotype. Adrenal phenotype in ΔCat and ΔCat;AdIgf2 mice was evaluated by haematoxylin & eosin staining (a–d) and by immunohistochemistry for Dab2 (e–h), Akr1b7 (i–l) and Ki67 (m–p). One column represents analysis of different sections from the same adrenal. Two ΔCat;AdIgf2 individuals showing the mildest (c, g, k, o) and worst (d, h, l, p) phenotypes were included. Black arrows show infiltrating mesenchymal subcapsular cells. White arrowheads show regions of nuclear pelomorphism. Black dashed lines in d demarcate areas of oedematous remodelling. **B**- **Analysis of proliferation. a**- Ki67 labelling-index was determined by counting the number of Ki67-positive cells over the total number of cells in five representative 40X fields per adrenal. Each individual index was plotted. The bar represents the mean index value for each group. ΔCat + ACTH and ΔCat;AdIgf2 + ACTH adrenals were obtained from 14 month-old ΔCat and ΔCat;AdIgf2 animals that were treated with ACTH for two months before culling. *P*-value was calculated using ANOVA followed by a post-hoc Fisher LSD test. **b-** Cyclin D1 expression was analysed by RTqPCR with cDNAs from wild-type, ΔCat, ΔCat;AdIgf2, ΔCat + ACTH and ΔCat;AdIgf2 + ACTH adrenals. Bars represent the mean relative quantification (Rq Tg/WT) of gene expression in at least 7 adrenals per genotype ± standard deviation. *P*-value was calculated using ANOVA followed by a post-hoc Fisher LSD test. **C- Determination of the Weiss score.** Weiss score was determined by a trained pathologist, using the criteria defined by Weiss for human adrenal tumours. The table only shows the criteria that were observed in our cohort. The table also includes the number of animals showing a Ki67 index above the critical 5% threshold as well as leukocyte infiltration and occurrence of oedematous remodelling. Statistical analysis was performed by a Fisher Exact Test. Exponent letters correspond to statistically significant differences (p<0.03) between control ΔCat and ΔCat;AdIgf2 adrenals (a), control and ACTH-treated ΔCat adrenals (b) and control and ACTH-treated ΔCat;AdIgf2 adrenals (c). Some values had to be considered as a group, to reach significance in the paired comparisons (brackets). **D- Histological analysis of ΔCat and ΔCat;AdIGF2 adrenals in ACTH-treated animals.** Twelve month-old ΔCat and ΔCat;AdIgf2 mice were treated for two months with ACTH before culling. Their adrenals were then analysed by haematoxylin & eosin staining (a–g) and proliferation was evaluated by Ki67 staining (h–j). One column represents analysis of a single adrenal. Two ΔCat;AdIgf2 + ACTH individuals showing the mildest (b, e, g, i) and worst (c, f, j) phenotypes were included. Pictures in d, e, f and g represent high magnification details of the corresponding adrenals in a, b and c. White arrowheads show areas of nuclear pleomorphism. Black dashed lines demarcate areas of oedematous remodelling (g) and necrosis (f). M, medulla; Co, Cortex; F, fasciculata; G, glomerulosa. Scale bar is 80 µm.

We then evaluated a possible cooperation at later stages by analysing 14 month-old transgenic mice. At this stage, ΔCat mice showed quite homogenous signs of tumour progression. Cortical dysplasia was evident (Figure 4A, b) and the cortex was mostly composed of glomerulosa cells as shown by Dab2 staining (Figure 4A, f). Fasciculata differentiation, analysed by Akr1b7 immunohistochemistry (Figure 4A, j), was also altered when compared with wild-type (Figure 4A, i). However there were no obvious signs of malignant progression. Consistent with these histological observations, Ki67 labelling index (Figure 4A, n) was increased when compared with wild-type mice but only one individual exceeded the 5% threshold considered as a marker of malignancy in patients [38] (Figure 4B, a). Anatomo-pathological evaluation showed that 4/5 ΔCat adrenals had a Weiss score of 0 and 1/5 had a Weiss score of 1 resulting from a marked decrease in the number of clear cells and accumulation of compact cells (Figure 4C & Figure S7). Histological analysis of ΔCat;AdIgf2 adrenals showed a broader range of phenotypes. Compound transgenics had histological features reminiscent of 14 month-old ΔCat adrenals, characterised by massive cortical dysplasia (Figure 4A, c–d), accumulation of basophilic spindle-shaped cells (Fig 4Ac) and cortical and medullary invasion by Dab2-positive zona glomerulosa cells (Figure 4A, g–h). However, these features were generally exacerbated when compared with stage-matched ΔCat adrenals. Indeed, the majority of ΔCat;AdIgf2 adrenals (6/7) exhibited a Weiss score of 1 or above (Figure 4C, p = 0,01 for Weiss ≥ 1 in Fisher's exact test), mostly characterised by prominent anisokaryosis and the presence of large nucleoli (Figure 4A, d, inset). One adrenal exhibited more profound alterations with both nuclear pleomorphism and the presence of mitoses (Weiss 2, Figure 4A, d, h, l, p). Small areas of oedematous remodelling (2/7) and leukocyte invasion (2/7) were also observed. In the most advanced tumours, Dab2 (glomerulosa, Figure 4A, h) and Akr1b7 (fasciculata, Figure 4A, l) were virtually undetectable, indicating loss of functional differentiation. The presence of these more aggressive features was correlated with increased Ki67 labelling index, which exceeded 20% in 2/7 ΔCat;AdIgf2 adrenals (Figure 4A, p & Figure 4B, a). The relative acceleration of tumour progression in response to Igf2 overexpression was also associated with a significant increase in Cyclin D1 expression in ΔCat;AdIgf2 when compared with ΔCat adrenals (Figure 4B, b), but did not result in further deregulation of malignancy markers expression (Figure S6 compared with Figure 3D). Altogether, these observations suggested that Igf2 overexpression had a moderate but significant promoting effect on tumour progression in 14 month-old ΔCat mice. This was also confirmed in the independent ΔCat;Scc:Igf2 compound transgenic line (Figure S7).

Adrenal tumour patients only show IGF2 overexpression in carcinomas but not adenomas. We thus decided to mimic this situation by further inducing Igf2 expression in ΔCat;AdIgf2 adrenals, right before the onset of spontaneous aggressive tumour transition. For this, 12 month-old ΔCat and ΔCat;AdIgf2 transgenic mice were treated with ACTH for two months. This resulted in a further 3.5 fold increase of Igf2 expression in adrenals from ACTH-treated ΔCat;AdIgf2 mice when compared with untreated animals (27 fold when compared with non transgenic animals, Figure S3E). As expected, this treatment did not induce Igf2 expression in ΔCat mice (Figure S3E). Histological analysis showed that ACTH treatment resulted in marked cellular hypertrophy (Figure 4D, d–e) in both ΔCat and ΔCat;AdIgf2 adrenals. It also induced an exacerbation of the tumour phenotype in both genotypes. Indeed, all animals had at least a Weiss score of 2 (Figure 4C, p = 0,0022 for ΔCat *vs* ΔCat + ACTH; p = 0,0047 for ΔCat;AdIgf2 *vs* ΔCat;AdIgf2 + ACTH in Fisher's exact test). This score was associated with both accumulation of compact cells, marked nuclear pleomorphism and enlarged nucleoli in both genotypes (Figure 4D, d–e, insets, white arrowheads). However, all these features were much more obvious in ΔCat;AdIgf2 than in ΔCat adrenals. Large areas of oedematous remodelling (Figure 4D, b, c, f, g, dashed lines and inset in g) and leukocyte infiltration (Figure 4C) were also essentially observed in this group. One ACTH-treated ΔCat;AdIgf2 adrenal presented as an overt carcinoma *in situ* (Weiss 3) with diffuse architecture (Figure 4D, c) high grade nuclear alterations, oedematous remodelling (Figure 4D, f, dashed line) and large areas of necrosis (Figure 4D, f, inset). ACTH treatment also induced a significant increase in Ki67 labelling index in ΔCat adrenals compared with their untreated counterparts (Figure 4B, a). Although the increase in Ki67 index was not significant in the ΔCat;AdIgf2 group it resulted in a slight shift upwards (mean 15% vs 11%) and in one adrenal showing a 34% index. Despite these aggressive features, ACTH treatment resulted in a normalisation of Cyclin D1 expression in both ΔCat and ΔCat;AdIgf2 adrenals. From these experiments, we concluded that elevating Igf2 expression accelerated tumour progression and induced carcinoma in situ development in the context of constitutive β-catenin activation. However, ACTH also had an intrinsic effect on tumour progression in ΔCat adrenals even though Igf2 expression levels were not increased (Figure S3E).

## Discussion

Despite recent progress, the molecular mechanisms of malignant adrenal cortical tumour development still remain elusive. The growth factor IGF2 is overexpressed in almost all adrenal cortical carcinomas, suggesting that it may play a role in malignant adrenal tumour formation in patients [8]–[10], [39]. Here, we show that overexpression of Igf2 from 7 to 87 fold over basal levels does not result in adrenal tumour formation in transgenic mice over 14 months, despite a mild increase in cortical cell proliferation (Figures 1 & 2). This indicates that Igf2 alone may stimulate adrenal cortical proliferation but that it is not able to induce oncogenic transformation. This observation is consistent with previously published data, showing that a 4 to 6-fold elevation of serum IGF2 levels in PEPCK-IGF2 transgenic mice did not induce adrenal tumour development [23]. The absence of oncogenic activity of Igf2 in the adrenal gland of transgenic mice may seem surprising at first. However, most models of Igf2 overexpression do not develop tumours although they recapitulate developmental overgrowth alterations associated with BWS [21], [22]. Interestingly though, about 20% of transgenic mice with overexpression of IGF2 under the control of the MMTV (Mouse Mammary Tumour Virus) promoter develop lung tumours after 6 months of age. This proportion rises to about 70% after 18 months [40]. This suggests that in this particular tissue IGF2 may behave as a weak oncogene. Surprisingly, the same transgenic mice do not show tumour development in the mammary epithelium, despite a 50 to 100-fold increase in IGF2 expression in their mammary glands and an inhibition of apoptosis during involution [41]. Therefore the oncogenic potential of Igf2 seems tissue-specific. Igf2 signalling relies on the IGF1R receptor, which can mobilise multiple intracellular pathways, including PI3K/AKT and MAP Kinases [42]. One hypothesis that could account for tissue-specific activity of Igf2 is the coupling of IGF1R signalling with distinct pathways, depending on the cell type. In this respect, one interesting observation is that in various cell lines in culture, mobilisation of the Igf1 receptor by Igf2, Igf1 or insulin can stimulate Wnt signalling, through inhibition of GSK3β [33] or phosphorylation and activation of β-catenin [34]–[37]. These mechanisms were proposed to account for some of the oncogenic activities of Igf2 and would be particularly relevant for the adrenal, as we previously showed that β-catenin was a *bona fide* oncogene in the adrenal cortex [14]. However, our analysis of β-catenin expression by immunohistochemistry, and of Wnt target genes expression by RTqPCR, failed to show activation of the Wnt/β-catenin pathway in Igf2 transgenic adrenals (Figure 2). It is therefore tempting to speculate that the absence of oncogenic activity of Igf2 in the adrenal may result, at least in part, from its incapacity to activate canonical Wnt signalling in this particular tissue.

Although overexpression of Igf2 *per se* may have low oncogenic potential in transgenic mouse models, there is ample evidence that Igf2 can cooperate with other pathways to accelerate tumour progression. This has been particularly well established for Wnt-dependent carcinogenesis in the colon. Indeed, increasing Igf2 levels through LOI (heterozygous deletion of H19) or with a keratin 10 promoter increases the number and size of colon adenomas as well as malignant progression in APC^Min/+^ mice [43]–[45]. Conversely, the reduction of Igf2 expression resulting from deletion of the transcriptionally active paternal allele or from the use of a soluble form of the scavenger IGF2R receptor, is associated with reduced number of adenomas and decreased malignant progression [44], [45]. Similar observations were also made in hedgehog/patched-dependent medulloblastomas and rhabdomyosarcomas [46], [47]. In malignant adrenal cortical tumours, the second most frequent alteration after IGF2 overexpression is constitutive activation of β-catenin [11], [13]. Carcinomas that developed with low frequency in ΔCat mice never showed spontaneous overexpression of Igf2 (14 and unpublished observations). Here, we decided to mimic the genetic context of human ACC by mating ΔCat mice with both AdIgf2 and Scc:Igf2 transgenic mice. Our analysis initially focused on the comparison of 10 month-old ΔCat and ΔCat;AdIgf2 or ΔCat;Scc:Igf2 compound transgenics. At this stage, ΔCat mice only developed benign adrenal hyperplasia and dysplasia (14 and this manuscript). Analyses of proliferation, differentiation and expression of malignancy markers showed that overexpression of Igf2 in this context had no significant effect on tumour development (Figure 3). This suggests that Igf2 overexpression is dispensable in the early stages of adrenal tumour development, which is consistent with the low expression of IGF2 in human ACA [9]. We thus evaluated the effect of Igf2 at a later time-point by analysing 14 month-old ΔCat and compound transgenic adrenals. Although there was still heterogeneity in tumour presentation in both ΔCat and ΔCat;AdIgf2 adrenals at this stage, histo-pathological analysis showed an almost systematic acceleration of tumour progression in the adrenals that overexpressed Igf2 (Figure 4). Indeed, a vast majority (6/7) of the tumour tissue from ΔCat;AdIgf2 mice had a Weiss score of 1 whereas the majority of ΔCat adrenals (4/5) displayed features consistent with a Weiss score of 0. This acceleration was associated with a significant increase in Cyclin D1 expression in ΔCat;AdIgf2 *vs* ΔCat adrenals and in a large increase in Ki67 labelling index (>20%) in 2/7 adrenals. However, there was still no significant alteration of malignancy markers expression and no overt signs of malignant progression. Overall, these observations show that Igf2 has the potential to accelerate adrenal cortex tumourigenesis. However, this effect is only significant in already advanced lesions and does not induce adrenal carcinoma development even in aged animals.

The mechanisms accounting for the late involvement of Igf2 in adrenal tumour progression are unclear. Similar observations were made in medulloblastomas. Indeed, genetic studies in mouse, showed that Igf2 was dispensable at early pre-cancerous stages but that it was required at later stages of tumour progression [47]. In vitro studies on adrenal carcinoma cell lines with increased IGF2 expression showed that blocking IGF1R activity resulted in both decreased proliferation and induction of apoptosis, indicating that IGF2 was involved in the control of both processes [19], [20]. However, we have shown that proliferation and to some extent, malignant transformation, are independent of Igf2 expression in ΔCat adrenals [14]. This suggests that Igf2 overexpression may stimulate another essential process to accelerate tumour progression. Here, we show that Igf2 overexpression in the context of constitutive β-catenin activation results in marked anisokaryosis and appearance of multiple and enlarged nucleoli that are not seen in stage-matched ΔCat adrenals (Figure 4A). Nucleoli are associated with RNA translation, a process dependent on ribosome biogenesis and the protein S6 [48]. Interestingly we show that one of the readouts of Igf2 overexpression in the adrenal cortex is the phosphorylation of the ribosomal protein S6 (Figure 1B). It is therefore tempting to speculate that one of the effects of Igf2 overexpression in adrenal tumour progression is dependent on an increased translation capacity, resulting from phosphorylation and activation of the S6 protein. This seems particularly relevant in light of the recent finding that mTOR pathway is activated and involved in growth stimulation of paediatric adrenocortical carcinomas [49], tumour entities that are also associated with IGF2 overexpression [20].

One surprising finding of our study is the relatively modest effect of Igf2 overexpression on tumour progression in compound transgenics. One may argue that the levels of overexpression achieved in our models are insufficient to induce aberrant stimulation of the IGF signalling pathway. We attempted to overcome this issue by stimulating transgene expression with chronic ACTH exposure over 2 months. This resulted in a marked 27-fold elevation of Igf2 expression (Figure S3E), which was in the range of overexpression in ACC patients [9]. This resulted in overall aggravation of the tumour phenotype in ΔCat;AdIgf2 adrenals, with one tumour presenting as an overt carcinoma *in situ* (Weiss 3). Some ΔCat;AdIgf2 tumours were also characterised by large areas of oedematous remodelling. Although the significance of this finding is unclear, it is correlated with the observation of large cystic hemorrhagic adrenal lesions in BWS patients [50], suggesting that Igf2 overexpression is directly responsible for the development of oedematous alterations. Altogether, these observations suggest that further increasing the levels of Igf2 expression can accelerate tumourigenesis in the context of β-catenin activation. However, one caveat of these experiments is the observation that tumours in the ΔCat group switch from Weiss 0 or 1 to Weiss 2 and show a significant increase in Ki67 labelling index after ACTH treatment (Figure 4). One may argue that ACTH treatment may indirectly (through elevation of Akr1b7:Cre expression) or directly (through pathway cross-talks [51]) stimulate β-catenin activity. However, this is unlikely as there was no significant increase in Axin2 expression in ΔCat and ΔCat;AdIgf2 adrenals following ACTH treatment (Figure S8). Even though the role of ACTH on adrenal cells proliferation is still a controversial subject [52], our observations may thus reflect an intrinsic growth promoting effect of this hormone in this particular genetic setting. Interestingly, analysis of IGF pathway actors shows increased expression of IGFBP2 and of the IGF receptor IGF1R in AdIgf2, ΔCat and ΔCat;AdIGF2 adrenals following ACTH treatment (Figure S9). Increased expression of these two positive IGF signalling factors may account for increased proliferation in the different genetic backgrounds. This would be consistent with the observation that IGFBP2 promotes proliferation and cloning efficiency of Y1 adrenocortical cells in culture [53]. These observations further suggest that the growth promoting effect of ACTH could be dependent on increased IGF signalling, irrespective of the genetic context.

Collectively, our data show that IGF2 overexpression in the context of constitutive Wnt/β-catenin activation results in a moderate acceleration of tumour development but that it is not sufficient to trigger malignant tumour progression.

One interesting observation of our studies is the accumulation of Pod-1 positive subcapsular progenitor cells in the adrenal cortex of AdIgf2 transgenic mice (Figure 1). Adrenal cortex cell renewal normally occurs through the recruitment of mesenchymal subcapsular progenitor cells [54]. These are recruited by Sonic Hedgehog (Shh) produced by poorly differentiated steroidogenic cells, localised in the vicinity of adrenal zona glomerulosa. Upon stimulation by Shh, progenitor cells express the hedgehog receptor Patched 1 (Ptch1) and the transcription factor Gli1 [32], [55]. Our RTqPCR analyses show overexpression of Ptch1 and Gli1 as well as Pod1. This strongly suggests that Igf2 induces the recruitment of adrenal cells progenitors. A similar role has been attributed to Igf2 for cerebellar granule cell precursors [56], [57] and for the mesenchymal progenitors of chondrocytes and osteoblasts [58]. Surprisingly, in AdIgf2 adrenals, Shh expression is downregulated (Figure 1). This suggests that the effect of Igf2 on adrenal progenitor recruitment is independent of Hedgehog signalling. In the cerebellum, Igf2 signalling can cooperate with Hedgehog to favour recruitment of cerebellar granule cell precursors. However, although Igf2 signalling is required for Hedgehog activity, Igf2 activity is independent of smoothened [56]. This suggests a cascade of signalling events in which Hedgehog, through Ptch1 and Gli1, induces Igf2 expression, which in turn signals to progenitors to stimulate their recruitment. Consistent with this hypothesis, Igf2 has been shown to be downstream of Hedgehog signalling in pluripotent mesenchymal cells [24] as well as in medulloblastomas and rhabdomyosarcomas [46], [47]. Based on these observations, it is tempting to speculate that Igf2 overexpression in the adrenal cortex bypasses hedgehog signalling to trigger progenitor cells recruitment. The use of Akr1b7 regulatory regions makes it unlikely for Igf2 to be expressed in the same cells that normally express Shh or Gli1 in the adrenal cortex. Consistent with this idea, Igf2 seems to be mostly expressed in differentiated cells of the cortex in AdIgf2 adrenals (Figure 1D). However, Igf2 is a diffusible factor that may act in a paracrine manner in this context. Interestingly, our unpublished analysis of ΔCat mice showed increased expression of Ptc1 and Gli1 in the adrenal cortex. However, in contrast with AdIgf2 transgenics, this was accompanied by overexpression of Shh (A. Berthon, unpublished observations). This suggests that Wnt signalling may be situated at the top of the cascade involved in the recruitment of adrenal progenitors. These observations obviously have implications for normal adrenal homeostasis but also suggest that aberrant recruitment of progenitor cells, resulting from deregulated Wnt, Hedgehog or IGF signalling, may participate in the development of adrenal cortex tumours.

In conclusion, using two distinct transgenic mouse lines allowing overexpression of Igf2, we have shown that increased Igf2 expression is not oncogenic *per se* in the adrenal cortex. We have also shown that it has a significant but moderate effect on tumour progression in the context of β-catenin activation, suggesting that these two alterations are not sufficient to trigger malignant adrenocortical tumourigenesis. These observations raise the question of the efficacy of IGF signalling targeted therapies in the context of ACC. Further studies are warranted to broaden our understanding of the molecular mechanisms involved in the formation of adrenocortical carcinomas.

## Supporting Information

Figure S1 **Establishment of transgenic lines overexpressing Igf2. A- Transgene constructs.** The 0.5 akr1b7:Igf2 transgene was constructed by cloning the full length mouse Igf2 cDNA (mIgf2) downstream of the Akr1b7 promoter (−510/+44) and upstream of a 3.5 kb segment of Akr1b7 intragenic regions, spanning intron 1 to intron 2 (Akr1b7 intron 2.1). The 4.5 Scc:Igf2 transgene was constructed by cloning mouse Igf2 cDNA downstream of a 4.5kb segment of human P450SCC regulatory regions (hP450SCC promoter) pA: mini intron and polyadenylation signal of SV40 T antigen. **B- Founders and lines.** Transgene constructs were microinjected into pronuclei of fertilized oocytes and transferred to pseudopregnant females. The table summarizes the outcome of these experiments by showing the number and identification (Id) of founders. Transgene copy numbers were evaluated by southern-blotting.
(TIF)

Figure S2 **A- Igf1r is expressed in the adrenal. Expression of Igf1 receptor (igf1r) was evaluated by RT-PCR with cDNAs from wild-type (WT) and transgenic adrenals (AdIgf2).** The positive control was composed of a mix of cDNAs from adrenals, testes, ovaries, spleen and liver. RT-PCR for 36b4 was included as a normalization reference. **B- S6Kinase phosphorylation is increased in AdIgf2 adrenals.** Expression of the phosphorylated S6Kinase was analysed by immunohistochemistry in wild-type and AdIgf2 adrenals. Black arrows show infiltrating mesenchymal subcapsular cells that are negative for phospho-S6Kinase staining. White arrowheads show negative cells in the wild-type adrenals. **C- GATA4 expression is increased in AdIGF2 adrenals.** Expression of GATA4 was analysed by immunohistochemistry in wild-type and AdIgf2 adrenals. Black arrows show GATA4-positive infiltrating cells. **D- Relative positions of zona glomerulosa and zona fasciculata are maintained in AdIgf2 adrenals.** Expression of Akr1b7 (green, fasciculata) and Dab2 (red, glomerulosa) was detected by co-immunohistochemistry on sections from wild-type and AdIgf2 adrenals. M, medulla; F, Fasciculata; G, Glomerulosa. Scale bar is 80 µm.
(TIF)

Figure S3 **Characterisation of the adrenal phenotype in Scc:Igf2 transgenic mice. A- Expression levels of Igf2 in the two transgenic lines.** Igf2 expression was analysed by RTqPCR on cDNAs from 10 month-old wild-type, 0.5 AdIgf2 and Scc:Igf2 (10 and 14 month-old) adrenals. Bars represent the mean relative quantification (Rq AdIgf2/WT) of Igf2 expression for each tissue in at least 5 samples per genotype ± standard deviation. *P*-value was calculated using Student's *t*-test. **B- Igf2 signalling is increased in Scc:Igf2 adrenals.** Expression of the phosphorylated ribosomal protein S6 was analysed by immunohistochemistry in WT (a) and AdIgf2 (b) adrenals. **C- Effect of Igf2 overexpression on adrenal histology and differentiation.** Histology was analysed by haematoxylin & eosin staining in wild-type (a), 10 month-old (b) and 14 month-old Scc:Igf2adrenals. Black arrows show infiltrating mesenchymal subcapsular cells. **D- Adrenal progenitor cells markers are overexpressed in Scc:Igf2 adrenal.** Expression of the progenitor cells markers, Gli1 and Pod1 was analysed by RTqPCR on cDNAs from wild-type (WT) and Scc:Igf2 adrenals. Bars represent the mean relative quantification (Rq SccIgf2/WT) of gene expression for each marker in at least 7 adrenals per genotype ± standard deviation. *P*-value was calculated using Student's *t*-test. **E- Expression of Igf2 after ACTH induction.** Twelve month-old ΔCat, AdIgf2 and ΔCat;AdIgf2 transgenic mice were treated for two months with ACTH or vehicle (ctrl). Igf2 expression levels in the adrenals were analysed by RTqPCR. Levels of accumulation in each group are presented relative to wild-type untreated adrenals. Bars represent the mean relative quantification (Rq Tg/WT) of gene expression for each marker in at least 4 adrenals per genotype and per condition ± standard deviation. *P*-value was calculated using Student's *t*-test. M, medulla; F, Fasciculata; G, Glomerulosa. Scale bar is 80 µm.
(TIF)

Figure S4 **Expression of Dab2 in 10 month-old ΔCat and ΔCat;AdIgf2 adrenals.** Expression of the zona glomerulosa marker Dab2 was analysed by RTqPCR on cDNAs from wild-type (WT), ΔCat and ΔCat;AdIgf2 adrenals. Bars represent the mean relative quantification (Rq Tg/WT) of gene expression in at least 7 adrenals per genotype ± standard deviation. *P*-value was calculated using Student's *t*-test.
(TIF)

Figure S5 **Analysis of the adrenal phenotype in 10 month-old ΔCat;Scc:Igf2 mice.** Scc:Igf2 transgenic mice were mated with ΔCat mice to generate ΔCat;SccIgf2 compound transgenics. The adrenal phenotype was analysed by haematoxylin & eosin staining in 10 month-old animals. Two ΔCat;Scc:Igf2 individuals showing the mildest (c, d) and worst (e, f) phenotypes were included. Pictures in d and f and g represent high magnification details of the corresponding adrenals in c and e respectively. Black arrows show infiltrating mesenchymal subcapsular cells. White dashed lines demarcate areas of compact cells accumulation. Accumulation of these cells in over 75% of the adrenal is considered as 1 point in Weiss score determination. Scale bar is 80µm.
(TIF)

Figure S6 **Analysis of malignancy markers expression in 14 month-old ΔCat;AdIgf2 adrenals.** Expression of VegfA, Connexinα43 (cnx43) and Nov/Ccn3 was analysed by RTqPCR with cDNAs from 14 month-old wild-type (WT), ΔCat and ΔCat;AdIgf2 adrenals. Bars represent the mean relative quantification (Rq Tg/WT) of gene expression for each marker in at least 6 adrenals per genotype ± standard deviation. *P*-value was calculated using Student's *t*-test.
(TIF)

Figure S7 **Analysis of the adrenal phenotype in 14 month-old ΔCat;SccIgf2 mice.** The adrenal phenotype was analysed by haematoxylin & eosin staining in 14 month-old animals. Three differentΔCat;SccIgf2 adrenals showing the range of phenotypes are presented (b-h). Pictures in f, g and h show high magnification details of pictures in b, c and d respectively. The adrenal in b and f is composed of a majority of compact cells (Weiss 1) delineated by white dashed lines. The adrenal in c and g is mostly composed of a central macro-nodule of spongiocytic cells, but only shows mild accumulation of compact cells (Weiss 0). The adrenal in d and h shows multiple areas of nuclear pleomorphism (white arrowheads, Weiss 1) and lymphocytic invasion (black arrowheads). Please note that the ΔCat adrenal in a and e also shows nuclear pleomorphism (Weiss 1), although overall cortical organisation is preserved. Black arrows show infiltrating mesenchymal subcapsular cells. Scale bar is 80µm.
(TIF)

Figure S8 **Expression of Wnt target gene Axin2 in ΔCat and ΔCat;AdIgf2 adrenals after ACTH treatment.** Twelve month-old ΔCat and ΔCat;AdIgf2 transgenic mice were treated for two months with ACTH or vehicle (ctrl). Axin2 expression levels in the adrenals were analysed by RTqPCR. Levels of accumulation in each group are presented relative to wild-type untreated adrenals. Bars represent the mean relative quantification (Rq Tg/WT) of gene expression for each marker in at least 5 adrenals per genotype and per condition ± standard deviation. There was no statistical difference between groups using Student's *t* test.
(TIF)

Figure S9 **Expression of IGFBP2 and IGF1R in response to ACTH treatment.** Twelve month-old AdIf2, ΔCat and ΔCat;AdIgf2 transgenic mice were untreated or treated for two months with ACTH. IGFBP2 and IGF1R expression levels in the adrenals were analysed by RTqPCR. Levels of accumulation in each group are presented relative to wild-type untreated adrenals. Bars represent the mean relative quantification (Rq Tg/WT) of gene expression for each marker in at least 5 adrenals per genotype and per condition ± standard deviation. Statistical analysis was performed using Student's *t* test.
(TIF)
