# Supplementary figures and images for: Analysis of the Role of Igf2 in Adrenal Tumour Development in Transgenic Mouse Models
Source: PLoS One. 2012 Aug 28;7(8):e44171. doi: 10.1371/journal.pone.0044171 (PMC3429465; doi:10.1371/journal.pone.0044171)

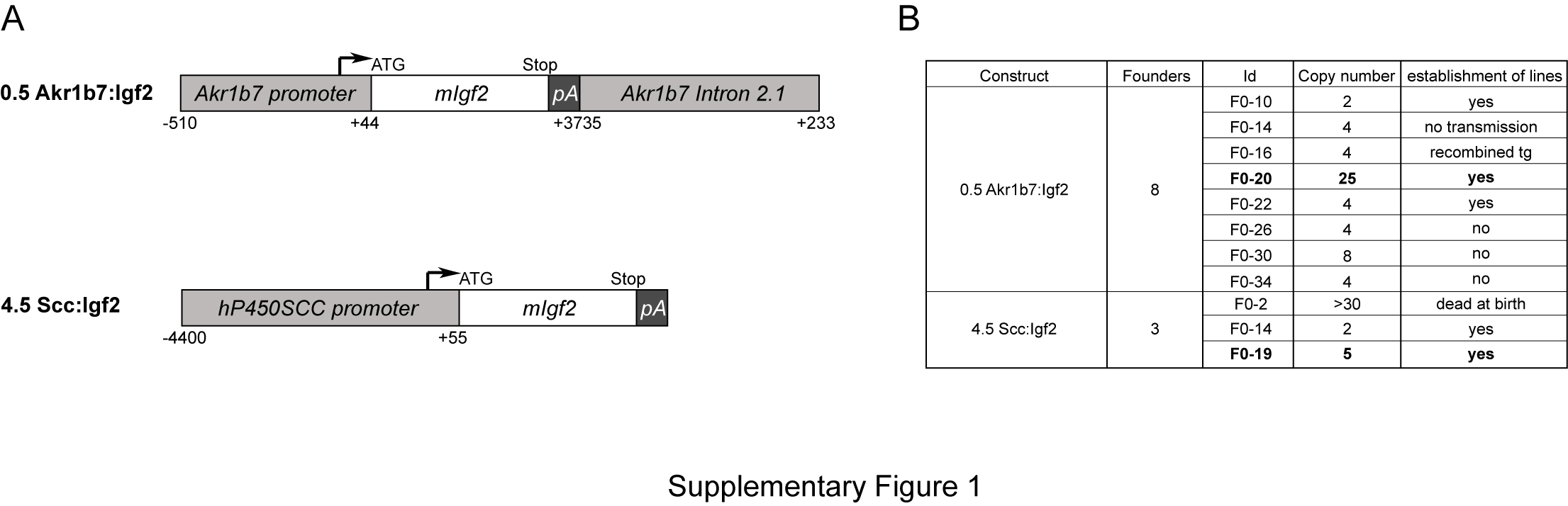

Supplement: Figure S1 — Establishment of transgenic lines overexpressing Igf2. A- Transgene constructs. The 0.5 akr1b7:Igf2 transgene was constructed by cloning the full length mouse Igf2 cDNA (mIgf2) downstream of the Akr1b7 promoter (−510/+44) and upstream of a 3.5 kb segment of Akr1b7 intragenic regions, spanning intron 1 to intron 2 (Akr1b7 intron 2.1). The 4.5 Scc:Igf2 transgene was constructed by cloning mouse Igf2 cDNA downstream of a 4.5kb segment of human P450SCC regulatory regions (hP450SCC promoter) pA: mini intron and polyadenylation signal of SV40 T antigen. B- Founders and lines. Transgene constructs were microinjected into pronuclei of fertilized oocytes and transferred to pseudopregnant females. The table summarizes the outcome of these experiments by showing the number and identification (Id) of founders. Transgene copy numbers were evaluated by southern-blotting. (TIF) [file pone.0044171.s001.tif]

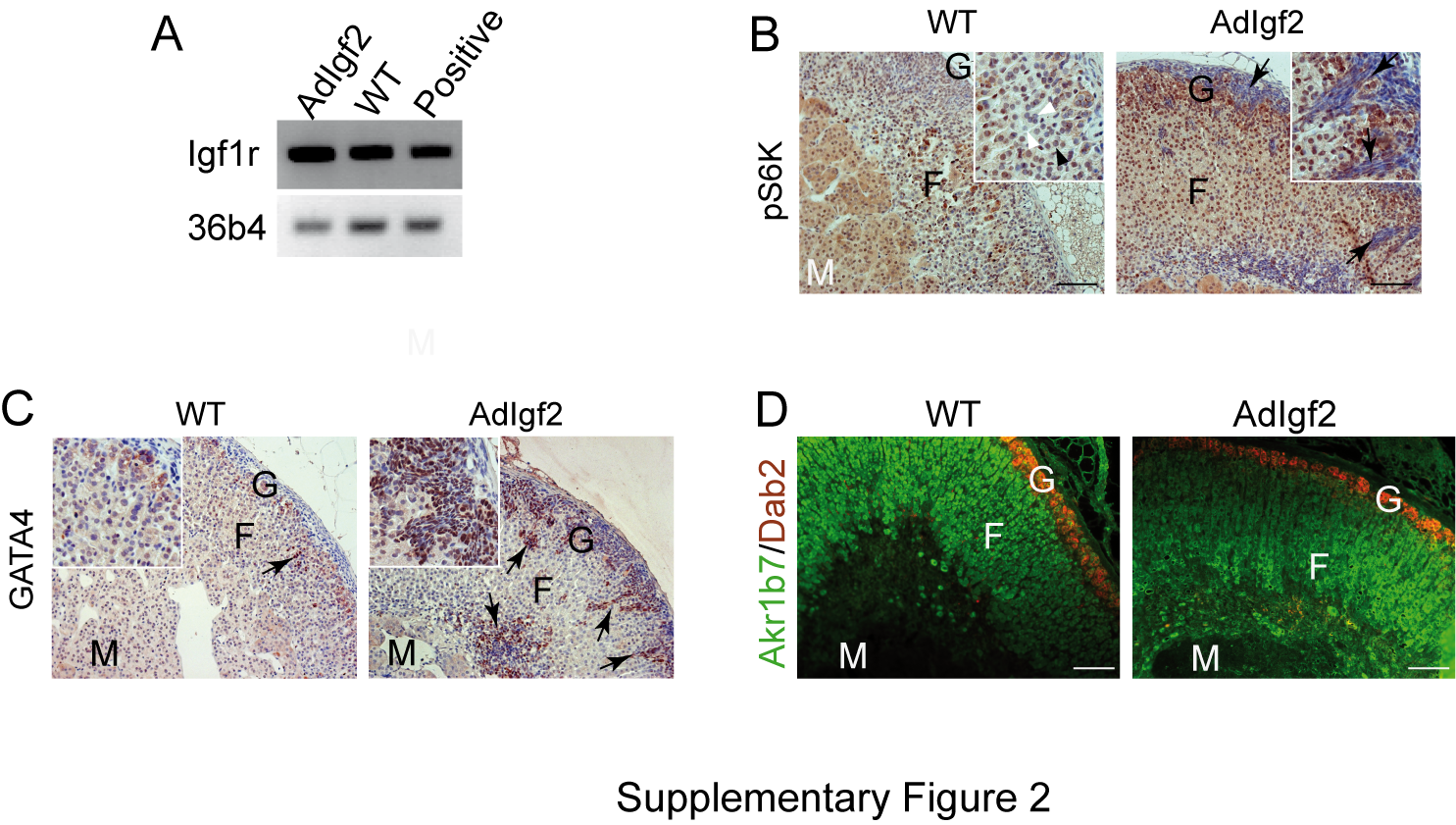

Supplement: Figure S2 — A- Igf1r is expressed in the adrenal. Expression of Igf1 receptor (igf1r) was evaluated by RT-PCR with cDNAs from wild-type (WT) and transgenic adrenals (AdIgf2). The positive control was composed of a mix of cDNAs from adrenals, testes, ovaries, spleen and liver. RT-PCR for 36b4 was included as a normalization reference. B- S6Kinase phosphorylation is increased in AdIgf2 adrenals. Expression of the phosphorylated S6Kinase was analysed by immunohistochemistry in wild-type and AdIgf2 adrenals. Black arrows show infiltrating mesenchymal subcapsular cells that are negative for phospho-S6Kinase staining. White arrowheads show negative cells in the wild-type adrenals. C- GATA4 expression is increased in AdIGF2 adrenals. Expression of GATA4 was analysed by immunohistochemistry in wild-type and AdIgf2 adrenals. Black arrows show GATA4-positive infiltrating cells. D- Relative positions of zona glomerulosa and zona fasciculata are maintained in AdIgf2 adrenals. Expression of Akr1b7 (green, fasciculata) and Dab2 (red, glomerulosa) was detected by co-immunohistochemistry on sections from wild-type and AdIgf2 adrenals. M, medulla; F, Fasciculata; G, Glomerulosa. Scale bar is 80 µm. (TIF) [file pone.0044171.s002.tif]

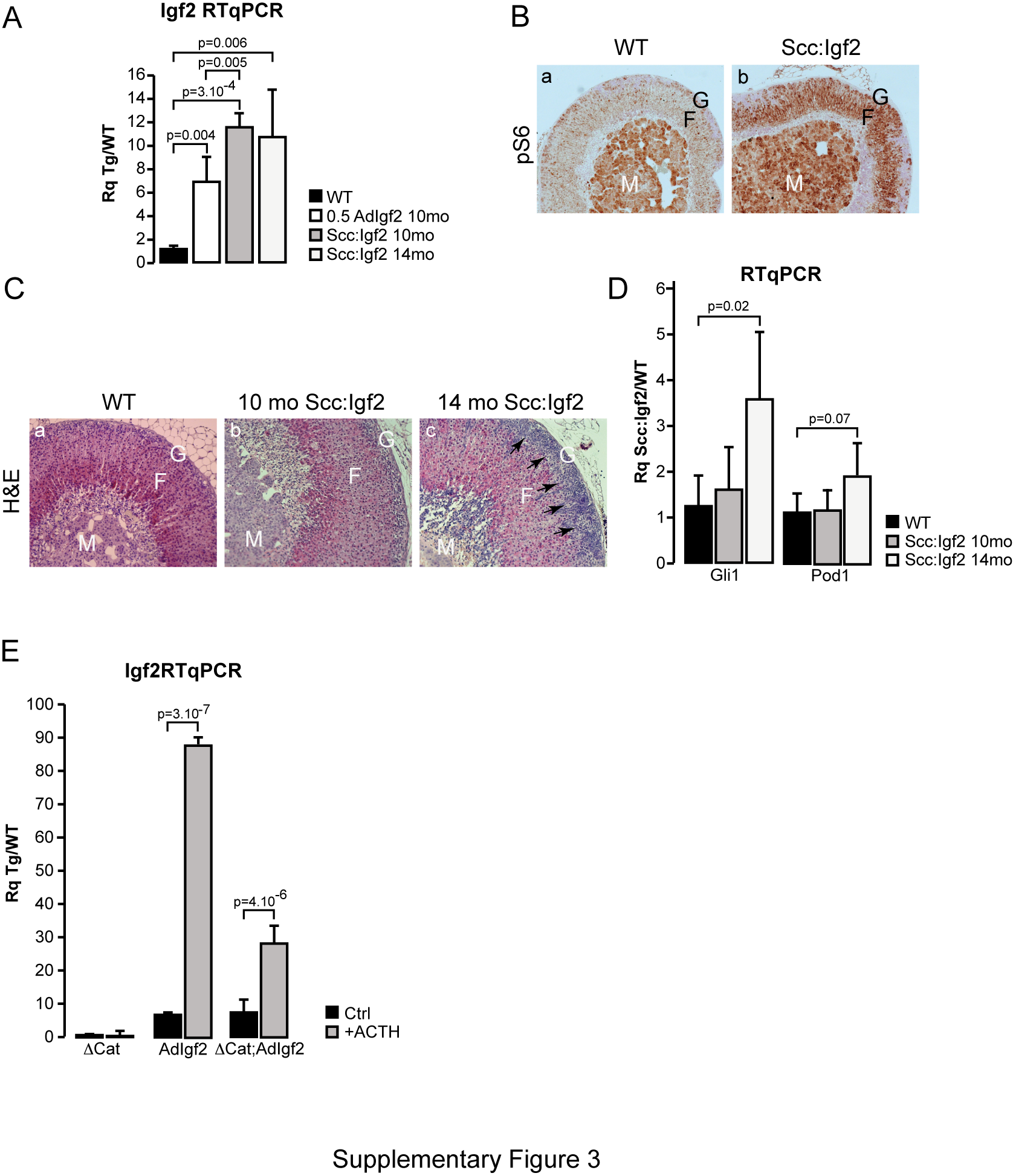

Supplement: Figure S3 — Characterisation of the adrenal phenotype in Scc:Igf2 transgenic mice. A- Expression levels of Igf2 in the two transgenic lines. Igf2 expression was analysed by RTqPCR on cDNAs from 10 month-old wild-type, 0.5 AdIgf2 and Scc:Igf2 (10 and 14 month-old) adrenals. Bars represent the mean relative quantification (Rq AdIgf2/WT) of Igf2 expression for each tissue in at least 5 samples per genotype ± standard deviation. P-value was calculated using Student's t-test. B- Igf2 signalling is increased in Scc:Igf2 adrenals. Expression of the phosphorylated ribosomal protein S6 was analysed by immunohistochemistry in WT (a) and AdIgf2 (b) adrenals. C- Effect of Igf2 overexpression on adrenal histology and differentiation. Histology was analysed by haematoxylin & eosin staining in wild-type (a), 10 month-old (b) and 14 month-old Scc:Igf2adrenals. Black arrows show infiltrating mesenchymal subcapsular cells. D- Adrenal progenitor cells markers are overexpressed in Scc:Igf2 adrenal. Expression of the progenitor cells markers, Gli1 and Pod1 was analysed by RTqPCR on cDNAs from wild-type (WT) and Scc:Igf2 adrenals. Bars represent the mean relative quantification (Rq SccIgf2/WT) of gene expression for each marker in at least 7 adrenals per genotype ± standard deviation. P-value was calculated using Student's t-test. E- Expression of Igf2 after ACTH induction. Twelve month-old ΔCat, AdIgf2 and ΔCat;AdIgf2 transgenic mice were treated for two months with ACTH or vehicle (ctrl). Igf2 expression levels in the adrenals were analysed by RTqPCR. Levels of accumulation in each group are presented relative to wild-type untreated adrenals. Bars represent the mean relative quantification (Rq Tg/WT) of gene expression for each marker in at least 4 adrenals per genotype and per condition ± standard deviation. P-value was calculated using Student's t-test. M, medulla; F, Fasciculata; G, Glomerulosa. Scale bar is 80 µm. (TIF) [file pone.0044171.s003.tif]

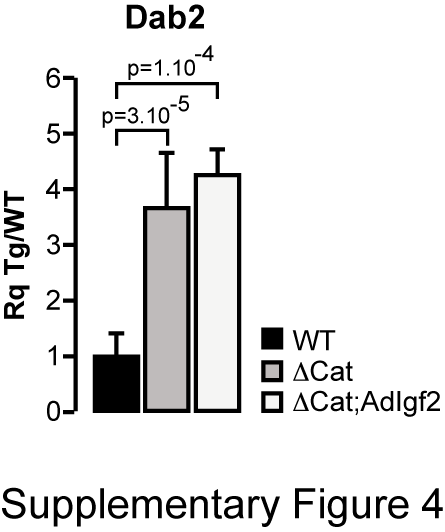

Supplement: Figure S4 — Expression of Dab2 in 10 month-old ΔCat and ΔCat;AdIgf2 adrenals. Expression of the zona glomerulosa marker Dab2 was analysed by RTqPCR on cDNAs from wild-type (WT), ΔCat and ΔCat;AdIgf2 adrenals. Bars represent the mean relative quantification (Rq Tg/WT) of gene expression in at least 7 adrenals per genotype ± standard deviation. P-value was calculated using Student's t-test. (TIF) [file pone.0044171.s004.tif]

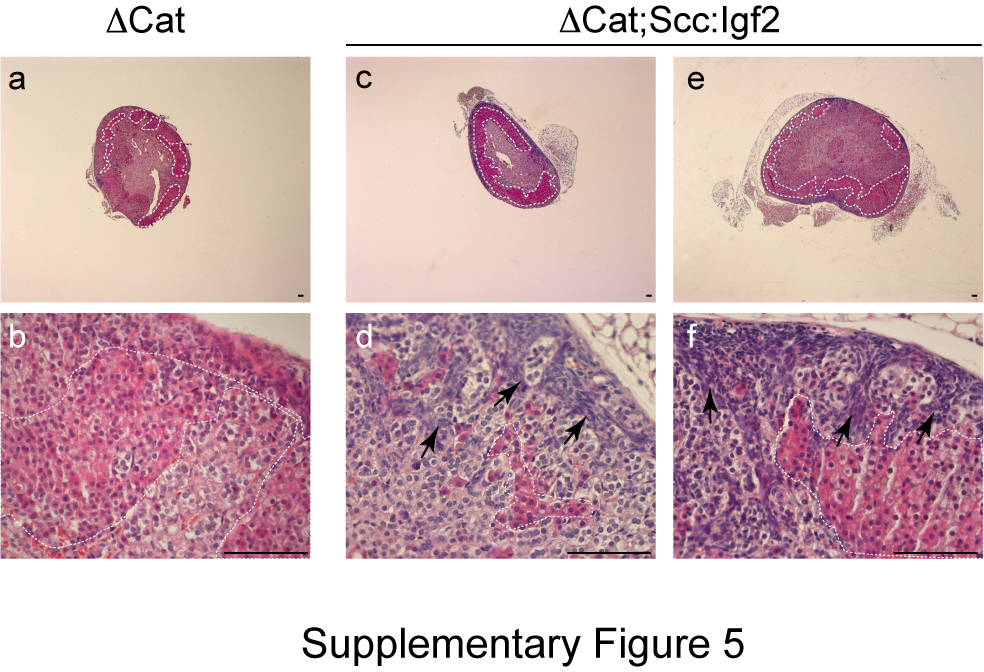

Supplement: Figure S5 — Analysis of the adrenal phenotype in 10 month-old ΔCat;Scc:Igf2 mice. Scc:Igf2 transgenic mice were mated with ΔCat mice to generate ΔCat;SccIgf2 compound transgenics. The adrenal phenotype was analysed by haematoxylin & eosin staining in 10 month-old animals. Two ΔCat;Scc:Igf2 individuals showing the mildest (c, d) and worst (e, f) phenotypes were included. Pictures in d and f and g represent high magnification details of the corresponding adrenals in c and e respectively. Black arrows show infiltrating mesenchymal subcapsular cells. White dashed lines demarcate areas of compact cells accumulation. Accumulation of these cells in over 75% of the adrenal is considered as 1 point in Weiss score determination. Scale bar is 80µm. (TIF) [file pone.0044171.s005.tif]

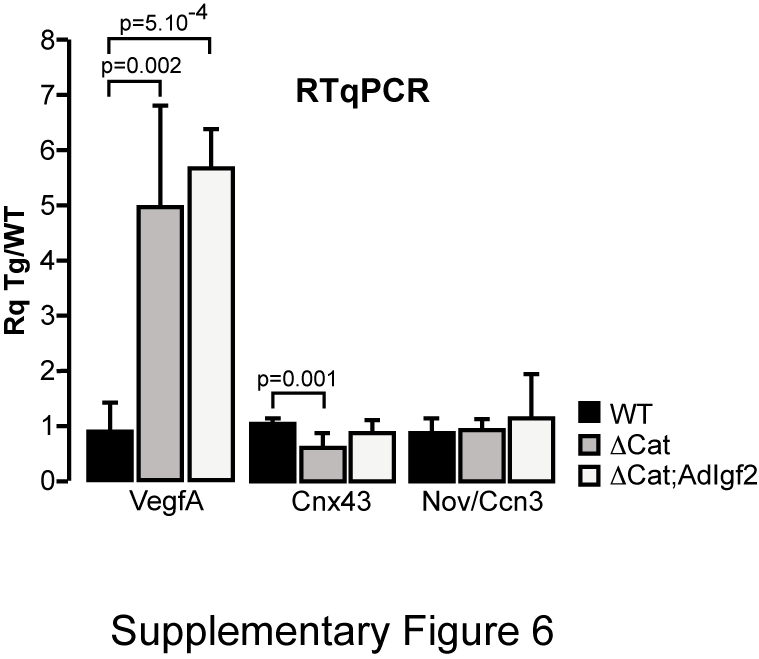

Supplement: Figure S6 — Analysis of malignancy markers expression in 14 month-old ΔCat;AdIgf2 adrenals. Expression of VegfA, Connexinα43 (cnx43) and Nov/Ccn3 was analysed by RTqPCR with cDNAs from 14 month-old wild-type (WT), ΔCat and ΔCat;AdIgf2 adrenals. Bars represent the mean relative quantification (Rq Tg/WT) of gene expression for each marker in at least 6 adrenals per genotype ± standard deviation. P-value was calculated using Student's t-test. (TIF) [file pone.0044171.s006.tif]

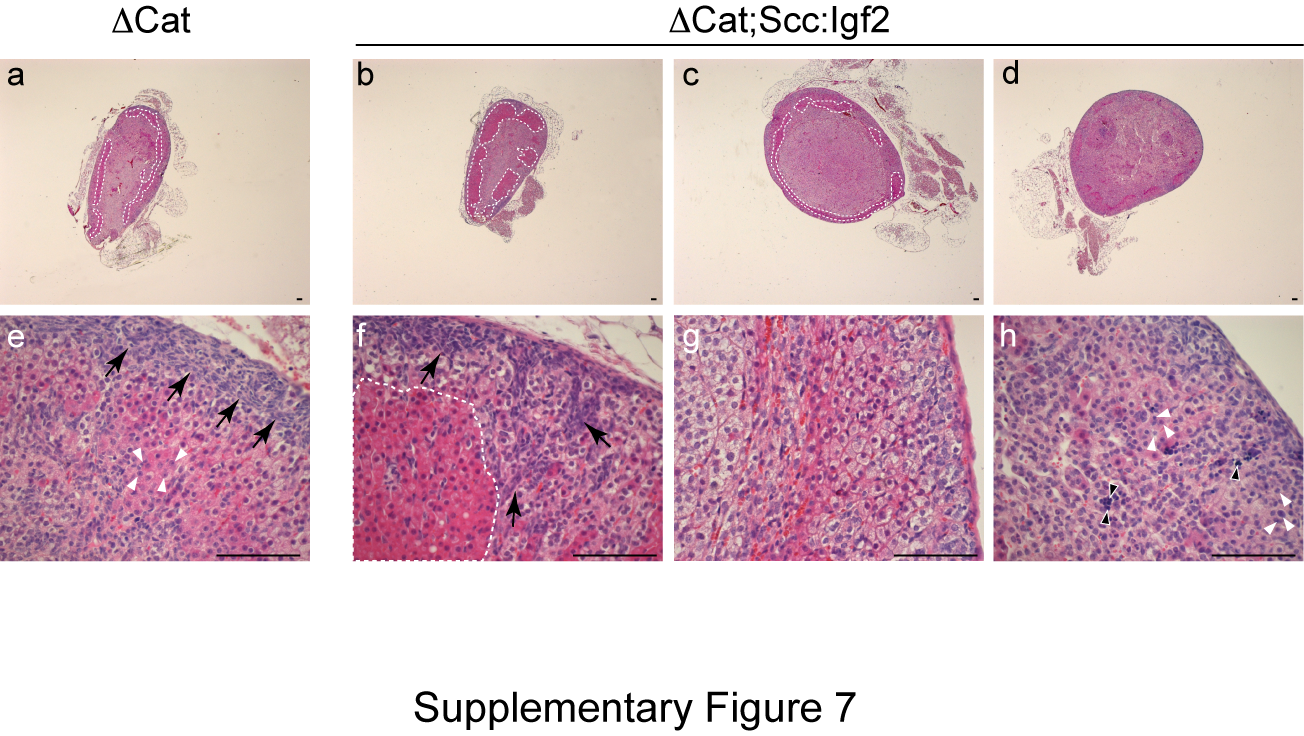

Supplement: Figure S7 — Analysis of the adrenal phenotype in 14 month-old ΔCat;SccIgf2 mice. The adrenal phenotype was analysed by haematoxylin & eosin staining in 14 month-old animals. Three differentΔCat;SccIgf2 adrenals showing the range of phenotypes are presented (b-h). Pictures in f, g and h show high magnification details of pictures in b, c and d respectively. The adrenal in b and f is composed of a majority of compact cells (Weiss 1) delineated by white dashed lines. The adrenal in c and g is mostly composed of a central macro-nodule of spongiocytic cells, but only shows mild accumulation of compact cells (Weiss 0). The adrenal in d and h shows multiple areas of nuclear pleomorphism (white arrowheads, Weiss 1) and lymphocytic invasion (black arrowheads). Please note that the ΔCat adrenal in a and e also shows nuclear pleomorphism (Weiss 1), although overall cortical organisation is preserved. Black arrows show infiltrating mesenchymal subcapsular cells. Scale bar is 80µm. (TIF) [file pone.0044171.s007.tif]

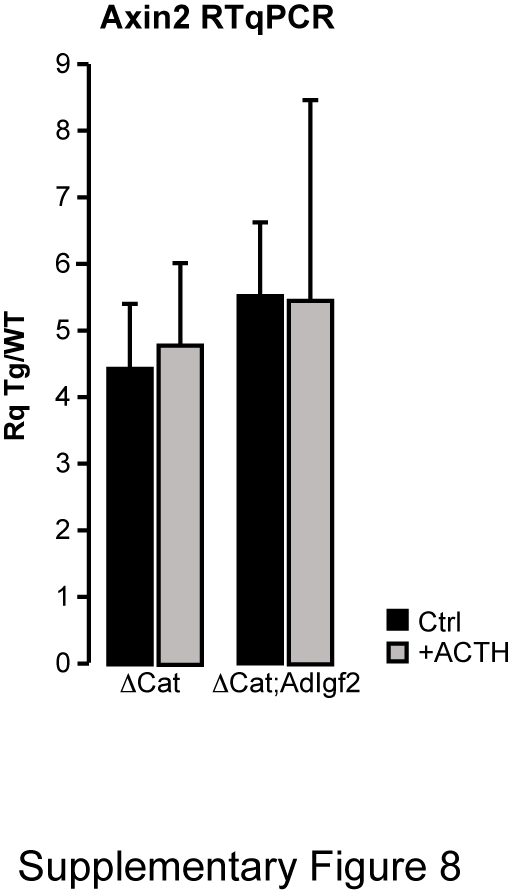

Supplement: Figure S8 — Expression of Wnt target gene Axin2 in ΔCat and ΔCat;AdIgf2 adrenals after ACTH treatment. Twelve month-old ΔCat and ΔCat;AdIgf2 transgenic mice were treated for two months with ACTH or vehicle (ctrl). Axin2 expression levels in the adrenals were analysed by RTqPCR. Levels of accumulation in each group are presented relative to wild-type untreated adrenals. Bars represent the mean relative quantification (Rq Tg/WT) of gene expression for each marker in at least 5 adrenals per genotype and per condition ± standard deviation. There was no statistical difference between groups using Student's t test. (TIF) [file pone.0044171.s008.tif]

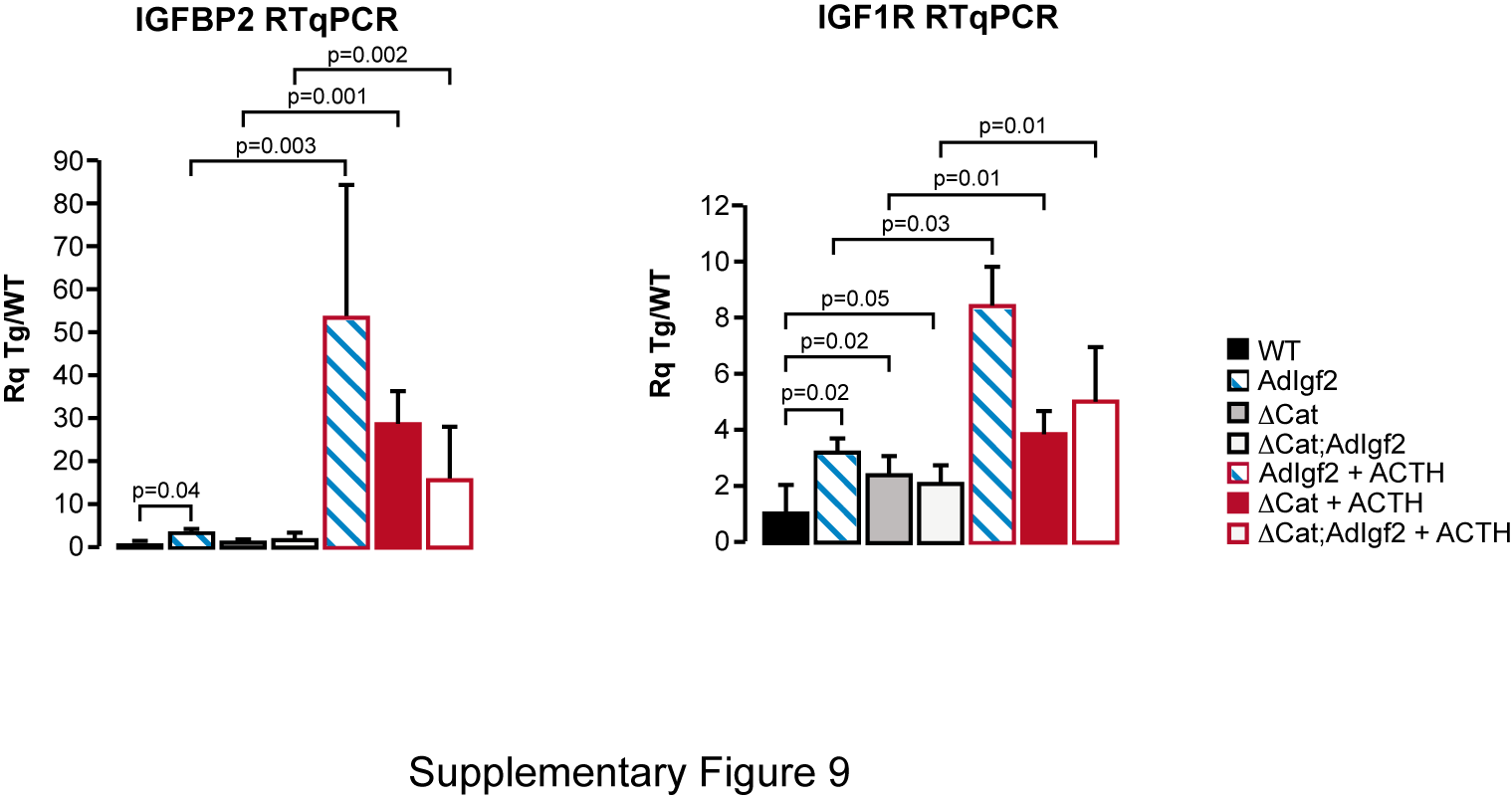

Supplement: Figure S9 — Expression of IGFBP2 and IGF1R in response to ACTH treatment. Twelve month-old AdIf2, ΔCat and ΔCat;AdIgf2 transgenic mice were untreated or treated for two months with ACTH. IGFBP2 and IGF1R expression levels in the adrenals were analysed by RTqPCR. Levels of accumulation in each group are presented relative to wild-type untreated adrenals. Bars represent the mean relative quantification (Rq Tg/WT) of gene expression for each marker in at least 5 adrenals per genotype and per condition ± standard deviation. Statistical analysis was performed using Student's t test. (TIF) [file pone.0044171.s009.tif]
